# PP2A activation targets MYCN in neuroblastoma

**DOI:** 10.1038/s41419-025-08253-0

**Published:** 2026-01-15

**Authors:** Nazia Nazam, Shamza Manzoor, Maryam Shaikh, Morgan L. Brown, Janet R. Julson, Colin H. Quinn, Swatika Butey, Sorina N. Shirley, Jamie M. Aye, Karina Yoon, Jianmei W. Leavenworth, Michael Ohlmeyer, Elizabeth A. Beierle

**Affiliations:** 1 Division of Pediatric Surgery, Department of Surgery, University of Alabama at Birmingham, Birmingham, AL USA; 2 Department of Neurosurgery, University of Alabama at Birmingham, Birmingham, AL USA; 3 Division of Pediatric Hematology Oncology, Department of Pediatrics, University of Alabama at Birmingham, Birmingham, AL USA; 4 Department of Cell Developmental and Integrative Biology, University of Alabama at Birmingham, Birmingham, AL USA; 5 Atux Iskay, LLC, Plainsboro, NJ USA

**Keywords:** Paediatric cancer, Paediatric cancer

## Abstract

Neuroblastoma (NBL) is the most common extracranial solid tumor of childhood, accounting for 7-10% of all children cancers, but leading to 15% of childhood cancer related deaths. Children with high-risk neuroblastoma (HR-NBL) lack effective treatments that achieve durable outcomes. While multiple factors stratify NBL patients into the high- risk category, *MYCN* amplification is a crucial determinant for that group. Thus far, efforts towards directly targeting MYCN have proven unsuccessful. Serine/threonine protein phosphatase 2 A (PP2A) functions as a tumor suppressor across cancers through its epigenetic effects, and its activity and tumor suppressor function are inhibited in NBL. We hypothesized that MYCN may be a target for PP2A, and that reactivation of PP2A may have a tumor suppressive effect on NBL. We employed studies to document the phenotypic, epigenetic, and in vivo effects of pharmacologic PP2A activation. Novel PP2A activators, ATUX-1215 or ATUX-5800, reduced *MYCN* mRNA abundance and MYCN phosphorylation and protein expression. PP2A activation decreased the acetylation of H3K27 (H3K27ac) as well as the enrichment of H3K27ac at the *MYCN* promoter. ATUX-1215 and ATUX-5800 treatment led to hypophosphorylation of RNA Pol II carboxy-terminal domain (CTD) and BRD4, transcriptional and epigenetic regulators respectively, coinciding with decreased MYCN expression and gene regulator acetylation. Tumor growth decreased in animals treated with ATUX-1215, and analysis of tumor specimens confirmed decreased MYCN expression. We conclude that pharmacologic PP2A reactivation may be a relevant therapeutic component in NBL treatment through its targeting of MYCN.

## Introduction

Neuroblastoma (NBL) is derived from precursor or immature cells of the sympathetic nervous system and is one of the most diffuse and highly malignant childhood cancers. As of 2021, an estimated 10,500 children in the US were diagnosed annually with cancer, of which 6% were NBL [1]. Significant advances have been made towards improving outcomes for patients with NBL, yet high-risk NBL (HR-NBL) continues to show an aggressive development pattern and dismal prognosis with a 5-year survival not surpassing 50%, compared to low and intermediate-risk patients in whom survival reaches 85-90%. This sub-set of NBL patients have limited availability of new therapeutic options and practically none that result in long-term survival, providing a stimulus to develop novel therapeutics to improve patient outcomes.

While multiple features stratify NBL patients into the high-risk category, *MYCN* amplification in the tumor is well established as the major factor leading to classification of a patient as having high-risk disease [2–4]. Aberrant MYCN levels due to enhanced transcription, protein stability, gene amplification and post-translational modifications (PTMs) confer MYCN pathogenicity [3, 5]. As an example, this single gene amplification is sufficient as an oncogenic driver to induce neoplastic transformation in neural crest- derived cells [4]. In this context, MYCN represents an ideal therapeutic target given its correlation with rapid tumor progression, poor prognosis and the limited expression in normal cells and tissue, suggesting high tolerability for a MYCN-targeted approach [3, 6]. However, due to the unstructured nature of *MYCN*, approaches that directly target this oncogene have been ineffective. Identifying alternative means to target MYCN are essential, and understanding the interplay between genetic and epigenetic factors may hold the key, as epigenetic modifications have been recognized as crucial determinants of *MYCN* levels in NBL development [7].

Serine/threonine protein phosphatase 2 A (PP2A) functions as a tumor suppressor across various cancers, and its activity is inhibited in several human malignancies including NBL [8, 9]. The tumor suppressive function of PP2A is attributed to its dephosphorylation potential towards numerous substrates involved in cell survival and growth [10–13]. Studies with an earlier tricyclic-sulfonamide PP2A activator, DBK-1154, suggested that these activators bind to the non-canonical PP2A-AC complex associated with RNA pol II- integrator [14, 15]. Further, a recent finding indicated that PP2A, together with IntS6/8 integrator protein, dephosphorylates residues within the RNA Pol (R Pol) II CTD that prevents productive transcriptional elongation, thus playing a crucial role in transcriptional repression [14].

Other investigations suggest that PP2A negatively regulates proteins that contribute to histone acetylation [16, 17], where a loss of PP2A function leads to an increase in histone acetylation, resulting in chromatin relaxation and subsequent gene transcription. In recent years, manipulating epigenetic modifiers, such as BET protein family members involved in *MYCN* activation, has proven successful in indirectly limiting its expression [18, 19]. Correspondingly, previous studies highlight hyperphosphorylation of the BET protein, bromodomain-containing 4 (BRD4), to be associated with PP2A inhibition, with a high occupancy of BRD4 and H3K27ac reported in aberrantly upregulated oncogenes such as *MYCN* [20, 21], prompting us to investigate whether PP2A activation would affect BRD4 protein and its ability to act as co-factor acetylating H3K27 and affecting *MYCN* [16, 22, 23].

The effects on cancer cell growth, the previous results on the potential for PP2A to target MYC family members [24, 25] and MYCN [8, 9, 26, 27], specifically, and the effects on transcriptional regulation of oncogenes, provide a foundation for the current study.

The current study contributes to the understanding of how pharmacologic activators of PP2A may regulate MYCN, potentially by reduced phosphorylation of RNA Pol II carboxy-terminal domain (CTD) and BRD4 which coincide with decreased acetylation at the promoter region of the *MYCN* gene, and by decreased phosphorylation and expression of MYCN protein.

## Materials and methods

### Cell lines, PDXs and culture conditions

Established NBL cell lines used in the present study, SK-N-AS (*MYCN* non-amplified; CRL-2137, female) and SK-N-BE(2) (*MYCN* amplified, CRL-2271, male) were obtained from the American Type Culture Collection (ATCC, Manassas, VA, USA). These cells were maintained under standard cell culture conditions (5% CO_2_, 37 °C). SK-N-AS cells were cultured in Dulbecco’s modified Eagle’s medium (DMEM, Corning Inc., Corning, NY, USA) supplemented with 10% fetal bovine serum (FBS, Hyclone, Suwanee, GA, USA), 1 μg/mL penicillin/streptomycin (Sigma–Aldrich, Burlington, MA, USA), 4 mM L-glutamine (Thermo Fisher Scientific Inc., Waltham, MA, USA), and 1 μM non-essential amino acids (Life Technologies, Carlsbad, CA, USA). SK-N-BE(2) cells were maintained in a 1:1 mixture of minimum Eagle medium (MEM) and Ham F-12 medium (Corning) with 10% FBS (Hyclone), 1 μg/mL penicillin/streptomycin (Sigma–Aldrich), 2 mM/L l-glutamine (Thermo Fisher Scientific Inc.), and 1 μM non-essential amino acids (Life Technologies).

The isogenic *MYCN* cells SH-EP (non-amplified) and WAC(2) (overexpressing) were generously provided by Dr. M. Schwab (Deutsches Krebsforschungszentrum, Heidelberg, Germany). These cells have been described in detail previously [28]. Briefly, the parent cell line, SH-EP, is a *MYCN* non-amplified cell line was stably transfected with a vector containing *MYCN* to create the WAC(2) MYCN overexpressing neuroblastoma cell line. These two cell lines were maintained in RPMI 1640 medium (Cytiva, Marlbourough, MA, USA) supplemented with 10% FBS (Hyclone), 1 μg/mL penicillin/streptomycin (Sigma–Aldrich), 4 mM L-glutamine (Thermo Fisher Scientific Inc), and 1 μM non-essential amino acids (Life Technologies). All cell lines were verified within the last 12 months using short tandem repeat (STR) analysis (Genomics Core, University of Alabama at Birmingham (UAB), Birmingham, AL, USA) and were deemed free of Mycoplasma infection (Universal Mycoplasma Detection Kit, 30-1012 K, ATCC).

The PDXs used in this study were *MYCN* amplified COA3 and COA6 and non-amplified COA129 which have been previously described [29]. These PDXs were generated at our institution under UAB Institutional Review Board (IRB) and Institutional Animal Care and Use Committee (IACUC) approved protocols (IRB-130627006, IACUC-009186, respectively). In brief, following written informed consent of the patients’ guardians, a fresh tumor specimen was obtained from surgical excision and temporarily placed in serum-free Roswell Park Memorial Institute 1640 (RPMI, 30–2001, ATCC) medium. For tumor implantation, NOD/SCID mice were anesthetized with 3% inhalational isoflurane and 16 mm^3^ tumor pieces were placed into the subcutaneous space of the flank. Animals were housed in a pathogen free environment and monitored routinely for overall health and tumor growth through weekly tumor volume measurements. Tumor volumes were calculated using the formula (width^2^ × length)/2, where width is the smaller measurement. When tumor volumes reached 2000 mm^3^, tumors were harvested, and dissociated using a Tumor Dissociation Kit (Miltenyi Biotec, San Diego, CA, USA) per manufacturer’s protocol. The dissociated tumor cells were maintained overnight prior to experiments in culture at 37 °C and 5% CO2 in neurobasal (NB) medium (Life Technologies) with the addition of B-27 without Vitamin A (Life Technologies), N2 (Life Technologies), l-glutamine (2 mM, Thermo Fisher Scientific Inc.), epidermal growth factor (10 ng/mL, Miltenyi Biotec), fibroblast growth factor (10 ng/mL, Miltenyi Biotec), gentamicin (50 μg/mL, Thermo Fisher Scientific Inc.) and amphotericin B (250 μg/mL, Thermo Fisher Scientific Inc.). PDX cells were not serially passed in culture. Real-time qPCR was done routinely to ascertain the human and mouse DNA percentage contained in the PDX cells to confirm that the tumors did not harbor murine contamination (TRENDD RNA/DNA Isolation; TaqMan QPCR/Genotyping Core Facility, UAB, Birmingham, AL, USA). The PDX cells were verified within the last 12 months using STR analysis (Genomics Core, UAB).

### Antibodies and experimental therapeutics

The following primary antibodies were used for immunoblotting: MYCN (84406), phospho- MYCN-Ser62 (13748), vinculin (13901), BRD4 (13440), acetyl-Histone H3-Lys9 (H3K9ac, 9649), acetyl-Histone H3-Lys27 (H3K27ac, 8173) and Histone H3 (4499) from Cell Signaling (Danvers, MA, USA); RNA pol II CTD phospho Ser2 (61083) and RNA pol II CTD phospho Ser5 (61986) from ActiveMotif (Cambridge, MA, USA); RNA polymerase II (PA5-88090) and acetyl-Histone H3-Lys122 (H3K122ac, PA5-105108) from Thermo-Fischer Scientific/Invitrogen; GAPDH (MAB374) from Sigma–Aldrich; β-actin (A5441) and phospho-Brd4-Ser492/Ser494 (ABE1453) from Millipore Sigma.

The diarylmethyl-4-aminotetrahydropyran sulfonamides, ATUX-1215 and ATUX-5800, were prepared by the methods disclosed in Ohlmeyer patent application WO2023/023594. Structures of these compounds and their pharmacokinetic and pharmacodynamic data have been reported [15, 30]. Compounds are characterized by NMR, MS and LC and are >98% purity. Details of structure-activity relationships will be reported elsewhere. In vitro microsome stability studies were carried out by Eurofins, Saint Louis, MO, USA. In vivo mouse pharmacokinetic studies were carried out by Charles River Laboratories, Worcester, MA, USA [30].

### Protein phosphatase 2A (PP2A) activation assay

SK-N-AS, SK-N-BE(2), or COA3 (1 × 10^6^) cells were treated with ATUX-1215 or ATUX-5800 (0-20 μM) for 4 hours followed by cell lysis using NP-40 lysis buffer (pH 7.9). PP2A activity was measured using the PP2A Immunoprecipitation Phosphatase Assay Kit (17- 313, Millipore Sigma) as per manufacturer’s protocol, with previously reported optimizations [8, 27]. Briefly, protein lysates were incubated with PP2A antibody at 4 °C with continuous rotation for 2 hours. Following the addition of assay buffers and malachite green solution, the plate was read at an absorbance of 650 nm using a microplate reader (Epoch Microplate Spectrophotometer, BioTek Instruments, Winooski, VT, USA). Phosphatase activity was extrapolated from a standard curve. Data are reported as fold change ± standard error of the mean (SEM) compared to control.

### Cell viability

An alamarBlue assay (Thermo Fisher Scientific, Inc.) assessed cell viability following treatment with ATUX-1215 or ATUX-5800. NBL cells (1.5 × 10^4^) were plated in 96-well plates and treated with ATUX-1215 or ATUX-5800 (0-80 μM). For viability assessment in PDXs, 3 × 10^4^ (COA6, COA3) or 5 × 10^4^ (COA129) cells were treated with 0-20 μM concentrations of ATUX-1215 or ATUX-5800. AlamarBlue dye (10 μL) was added after 24 hours (established cell lines) and the plates were read in a microplate reader (Epoch Microplate Spectrophotometer) for absorbance at 570 nm, using 600 nm as a reference wavelength. Since PDX cells turned the dye over more slowly, after adding alamarBlue dye (10 μL), plates were read for absorbance at 72 hours. Experiments were completed in biologic triplicates, viability reported as fold change ± SEM and the respective lethal dose 50% (LD_50_) was calculated.

### Colony forming assay/clonogenic assay

Clonogenic assay was performed by seeding SK-N-AS and SK-N-BE(2) cells in a 6-well plate at low density (1000 for SK-N-AS and 800 for SK-N-BE(2)) followed by treatment with either ATUX-1215 or ATUX-5800 (0, 2.5, 5, 7.5 μM in SK-N-AS and 0, 5, 10, 15 μM in SK-N-BE(2)). Plates were maintained in culture at 37 °C in 5% CO2 for 14 days when cells were fixed and stained with 0.5% crystal violet in 10% methanol. The plates were scanned and colonies quantified using ImageJ software (https://imagej.net/ij/, Ver 1.49, accessed October 2023).

### Cell motility assays

A modified Boyden chamber assay was employed to determine motility. We used 6.5 mm Transwell inserts with 8 µM pore polycarbonate membranes (Corning Inc.) in 24-well culture plates. The bottoms of the inserts were coated with human laminin (10 μg/mL, AG56P, Millipore Sigma). For invasion, the inserts were additionally coated on top with Matrigel (1 mg/mL, BD Biosciences, Franklin Lakes, NJ, USA). SK-N-AS and SK-N-BE(2) cells were pretreated with ATUX-1215 or ATUX-5800 (0, 10 and 20 μM) for 24 h and then 5 × 105 cells plated onto the top of the insert. Doses were below LD_50_ concentrations keeping in mind non-viable cells will not move. The insert was then placed into a well containing 300 μL of 10% FBS (Hyclone) supplemented conditioned media derived from respective NBL cell lines. After 24 h, the cells on top of the inserts were removed using a cotton swab. The inserts were fixed in 4% paraformaldehyde prior to staining with crystal violet. The Laxco SeBa digital microscope system (Laxco Inc., Mill Creek, WA, USA) was used to take images of the inserts at predetermined locations at 40× and then cells were quantified using ImageJ software (Ver 1.49, accessed March 2023). Experiments were repeated with biologic triplicates and migration or invasion reported as mean fold change in number of cells ± SEM.

### Immunoblotting

Whole cell lysates were prepared in RIPA buffer (50 mM Tris-HCl, pH 7.4, 150 mM NaCl, 1 mM EDTA, 1% Triton X-100, 1% sodium deoxcycholate, 0.1% SDS) or histones extracts in Triton Extraction Buffer (TEB) buffer (PBS with 0.5% Triton X-100, 2 mM PMSF, 0.02% NaN_3_). Fractionated nuclear lysates were prepared in nuclear extraction buffer (5 mM HEPES, pH 7.9; 1.5 mM MgCl_2_.6H20; 300 mM NaCl; 0.5 mM DTT; 0.2 mM disodium EDTA; 26% glycerol). Protein extraction buffers were supplemented with protease inhibitors (Sigma–Aldrich), phosphatase inhibitors (Sigma–Aldrich), and phenyl-methane sulfonyl fluoride (Sigma Aldrich). Protein concentrations were determined using a Micro BCA Protein Assay Kit (Thermo Fisher Scientific, Inc.), separated by electrophoresis on SDS-PAGE gels, and transferred to Immobilon-P polyvinylidene fluoride (PVDF) transfer membrane (Millipore Sigma). Precision Plus Protein Kaleidoscope Standards molecular weight markers (161–0375, Bio-Rad, Hercules, CA, USA) and high range markers (12949S, Cell Signaling Technology) were used to confirm the expected size of target proteins. Antibodies were used in accordance with the manufacturers’ recommended protocol. Samples were visualized by enhanced chemiluminescence (ECL) using Luminata Classico and Luminata Crescendo Western horseradish peroxidase (HRP) substrates (Millipore Sigma). Anti-β-actin, -GAPDH or -vinculin were used as an internal control to ensure equal protein loading between samples.

### Quantitative real time PCR (RT-qPCR)

RT-qPCR assessed the mRNA abundance of *MYCN* in NBL cells. Briefly, total cellular RNA was extracted using the RNeasy kit (Qiagen, German Town, MD, USA) according to the manufacturer’s protocol and cDNA synthesized using an iScript cDNA Synthesis kit (Bio-Rad) according to supplier’s instructions. SsoAdvanced SYBR Green Supermix (Bio-Rad) was utilized according to manufacturer’s protocol. Primers specific for *MYCN* are as follows: (Forward: 5’- ACTGTAGCCATCCGAGGACA-3’, Reverse: 5’- CAAGCCCTGCTCCTTACCTC-3’) (Invitrogen). qPCR was performed with 10 ng cDNA in 20 μL reaction volume. An Applied Biosystems 7900HT cycler (Applied Biosystems, Foster City, CA, USA) performed the amplification with cycling conditions of 95 °C for 2 min, followed by 39-cycle amplification at 95 °C for 5 s and 60 °C for 30 s. All mRNA levels were analyzed in triplicate normalized to the level of *GAPDH* gene expression as an internal control. The ΔΔCt method calculated the gene expression [24], which was reported as mean fold change ± SEM.

### Chromatin immunoprecipitation (ChIP) and ChIP- qPCR

The ChIP assay was performed using the SimpleChIP Plus Sonication CHIP Kit (Cell Signaling Technology, #56383) per recommended protocol with some modifications. SK-N-BE(2) (3 × 10^7^ cells) input control (0 µM) or treated cells (10 and 20 µM of ATUX-1215 or ATUX-5800 for 24 h) were fixed in 1% formaldehyde at room temperature (RT) in a 15 cm culture dish for 10 min to allow cross-linking. The reaction was quenched by adding glycine (0.125 M) for 5 min. Fixed cells were washed with ice-cold PBS and scraped to harvest cell pellet. The nucleic fraction was isolated from the lysate by resuspending in the ChIP Sonication—Cell Lysis Buffer followed by the Nuclear Lysis Buffer. After fragmenting the nuclear fraction via sonication (Fisherbrand Model 50 Sonic Dismembrator, ThermoFisher Scientific), the chromatin was incubated with anti-H3K27ac ChIP Grade antibody (2 μg per reaction, rabbit polyclonal, AB4729; Abcam Inc., Waltham, MA, USA) or control rabbit IgG (2729 P; Cell Signaling Technology) at 4 °C with rotation overnight, resuspended in ChIP-Grade Protein G beads to each immunoprecipitation reaction, and incubated for another 2 h at 4 °C with rotation. Antibody/protein G beads were washed in low and high salt wash and proceeded for chromatin (DNA) elution. The captured protein-DNA was de-crosslinked with 5 M NaCl and Proteinase K at 65 °C for 2 h. Lastly, DNA was purified using spin columns per the manufacturer′s protocol.

### ChIP-qPCR

The immunoprecipitated DNA was checked for enrichment using RT-qPCR. Primer sets used for ChIP-qPCR-based validation of H3K27ac at *MYCN* promoter were:

Set 1-

TTGCTCAACGTTGGCCTCG (forward); TGCAATGCAGCACCCACCCT (reverse)

Set 2-

AGGGTGGGTGCTGCATTGCA (forward); AGGCTGCAAAGCTGGGAAGC (reverse).

ChIP qPCR was completed with three biologic replicates.

### Animal statement

The UAB IACUC (IACUC-09803) approved all animal experiments, and the studies were conducted within institutional, national, and NIH guidelines and in compliance with the Animal Research: Reporting of In Vivo Experiments (ARRIVE) guidelines.

### In vivo tumor growth

SK-N-BE(2) (1.5 × 10^6^) cells in 25% Matrigel (BD Biosciences) were injected into the right flank of 6-week-old female athymic nude mice (Fredricks, Charles River, Wilmington, MA, USA) (*n* = 7 for vehicle treated, *n* = 8 for ATUX-1215 and *n* = 9 for ATUX-5800 experimental groups). Calipers were used to measure tumors three times per week, and volumes were calculated by the formula (width^2^ × length)/2, where width was the smaller measurement. After tumors reached a volume of 100 mm^3^, animals were randomized using an online randomization tool (https://www.gigacalculator.com/randomizers/randomizer.php, accessed September 2024) to three groups to receive 100 µL of either vehicle (N,N-dimethylacetamide (DMA, 271012, Sigma–Aldrich) and Kolliphor HS 15 (Solutol, 42996, Sigma–Aldrich), ATUX-1215 or ATUX-5800 (75 mg/kg in DMA and Solutol) twice daily by oral gavage; dosing based on previously published in vivo cancer models [30]. Animals were weighed weekly and were humanely euthanized in their home cages with CO_2_ and cervical dislocation when control tumors reached 2000 mm^3^; either 14 days following treatment initiation, or when IACUC parameters were met. Power analysis was completed prior to initiating the study using power and sample size calculator online tool (https://www.gigacalculator.com/calculators/power-sample-size calculator.php#powerandsamplesize, accessed September 2024). Investigators were not blinded to the treatments.

### Immunohistochemistry

Formalin-fixed, paraffin-embedded animal tumor specimens were cut (6 μm sections), placed on positive slides, baked at 70 °C for 1 h, deparaffinized, rehydrated and steamed. Standard hematoxylin and eosin (H&E) staining was completed to identify the location of tumor cells. For IHC, slides were incubated with primary antibodies (anti- MYCN (84406), Cell Signaling Technologies; phospho-Brd4-Ser492/Ser494 (ABE1453), Millipore Sigma; Ki67, (ab11580), Abcam) at 1:100 dilution overnight at 4 °C. The slides were washed with PBS and rabbit secondary antibody (R.T.U. biotinylated universal antibody, Vector Laboratories, Burlingame, CA, USA) added for 30 minutes at 22 °C. Staining reaction was applied for 30 min at RT with VECTASTAIN Elite ABC reagent (PK-7100, Vector Laboratories) and Metal Enhanced DAB Substrate (Thermo Fisher Scientific) for 4 (MYCN), 5 (pBRD4), or 3.5 (Ki67) minutes. All slides were counterstained with hematoxylin. A negative control was included for each IHC run (rabbit IgG, 1 μg/mL, EMD Millipore, Burlington, MA, USA). IHC staining was quantified with ImageJ (https://imagej.net/ij/, Version 1.49, accessed, June-July, 2025). The investigator completing analysis of the staining was blinded to treatment groups.

### R2-database analysis for NBL dataset

R2 Genomics Analysis and Visualization Platform was accessed for mRNA expression data from primary human NBL samples (Kocak-649) to evaluate the association of *BRD4* gene expression with event free and overall survival in *MCYN* non-amplified and *MYCN* amplified NBL patient dataset [31].

### Statistical tools and analyses

Experiments were performed with at least three biologic replicates. Data reported as the standard error of the mean (SEM) as deemed appropriate. Densitometry of immunoblots was performed using ImageJ software (https://imagej.net/ij/, Version 1.49, accessed, October-December, 2024 and June-July, 2025).

## Results

### NBL cells are sensitive to PP2A activation irrespective of *MYCN* amplification

**Fig. 1:**
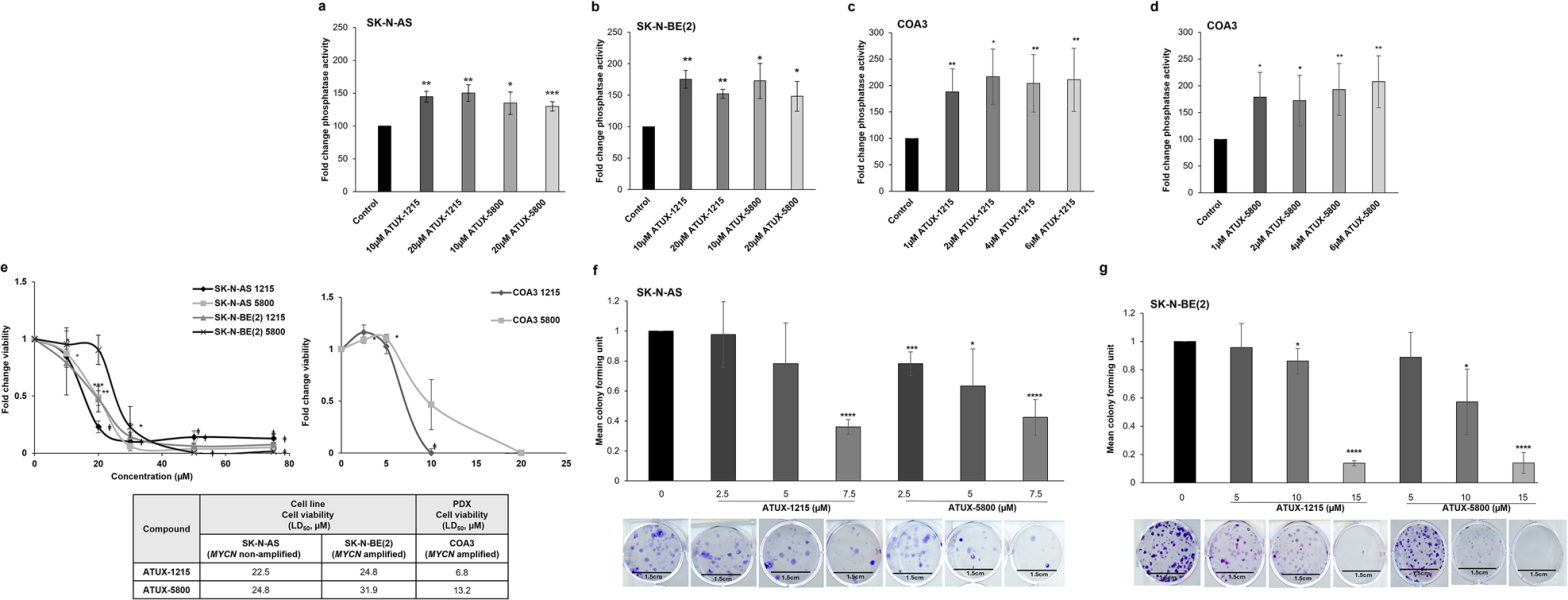
ATUX-1215 and ATUX-5800 activate PP2A and exert cytotoxic effect on NBL cells. **a**–**c** 1 × 10^6^ NBL cells were treated with ATUX-1215 or ATUX-5800 at increasing concentrations (0 (Control)–20 μM) for 4 h and PP2A activation was assessed. PP2A activity was significantly increased in SK-N-AS (20-50%, **a**), SK-N-BE(2) (40–70%, **b**), and COA3 PDX (70–90%, **c**, **d**) cells with either compound. e Established NBL and PDX cells were treated with increasing concentrations of ATUX-1215 or ATUX-5800 for 24 (SK-N-AS, SK-N-BE(2), 0–80 μM) or 72 h (COA3, 0–20 μM), and cell viability was measured using alamarBlue assay. Lethal dose 50% (LD_50_) concentrations were calculated using dose-response curves and range between 22.5 to 31.9 µM in NBL cell lines (**e**, left panel), and 6.8–13.2 µM in the COA3 PDX cells (**e**, right panel). Calculated LD_50_ data from three independent biologic replicates are presented in tabular form (**e**, *lower panel*). **f**, **g** Low cell numbers (1000 for SK-N-AS and 800 for SK-N-BE(2)) were treated with increasing concentrations of ATUX-1215 or ATUX-5800 to assess their effects on single cell proliferation. The cells were allowed to grow for 14 days, fixed and stained with crystal violet. Both compounds led to a significant inhibitory effect on the colony forming ability of SK-N-AS (**f**) and SK-N- BE(2) cells (**g**). For PP2A assay, data are normalized to control group (100), and for colony forming assay, control group (1). The data represent at least three independent biologic replicates and are presented as mean ± standard error of mean (SEM). Statistical comparison was completed with two-tailed Student’s *t* test. *p ≤ 0.05, **p ≤ 0.01, ***p ≤ 0.001, ****p ≤ 0.0001, ǂ*p* ≤ 0.00001. PP2A, protein phosphatase 2A; NBL, neuroblastoma; PDX, patient-derived xenograft; 1215, ATUX-1215; 5800, ATUX-5800.

We aimed to investigate the functional effect of small molecule-based PP2A reactivation in NBL. We first confirmed the levels of PP2A activation in *MYCN* non-amplified, SK-N-AS, and *MYCN* amplified, SK-N-BE(2) cell lines and *MYCN* amplified, COA3 PDX cells. The PP2A enzyme activation in SK-N-AS cells was significantly increased (20-50%) when treated with PP2A activators (ATUX-1215 or ATUX-5800) compared to control (Fig. 1a). Similarly, there was a significant increase (40-70%) in phosphatase activity in SK-N-BE(2) cells (Fig. 1b) and significant increase (70–90%) in the COA3 cells (Fig. 1c, d) following treatment with ATUX-1215 or ATUX-5800. We then assessed the effect of these PP2A activators on cell killing in SK-N-AS, SK-N-BE(2), SH-EP and WAC(2) cells and the human NBL PDXs, COA3, COA6, and COA129. Cells were treated with ATUX-1215 or ATUX-5800 at increasing concentrations for 24 h and cell viability was assessed using alamarBlue assay. The lethal dose 50% (LD_50_) was calculated based on the viability curves. We identified a LD_50_ of ATUX-1215 and ATUX-5800 of 22.5 and 24.8 µM, respectively, in SK-N-AS, and 24.8 and 31.9 µM, respectively, in SK-N-BE(2) cells (Fig. 1e, *left panel*). Treatment of COA3 PDX cells with these activators killed 50% cells at 6.8 and 13.2 µM for ATUX-1215 and ATUX-5800, respectively (Fig. 1e, *right panel*). LD_50_ values are presented in tabular form for SK-N-AS, SK-N-BE(2) and COA3 (Fig.1e, *lower panel*). The dose-response curves and summarized LD_50_ in SH-EP, WAC(2), COA6, and COA129 show similar responses (Fig. S1a–d). We then examined the effect of these PP2A activator molecules on NBL single cell clonogenicity. After 14 days, there was a significant inhibition in colony formation in ATUX-1215 or ATUX-5800 treated SK-N-AS and SK-N-BE(2) cells compared to untreated controls (Fig. 1f, g). These data indicate PP2A reactivation exerts a cytotoxic effect on NBL cells in vitro.

### PP2A activation inhibits NBL cell motility

**Fig. 2:**
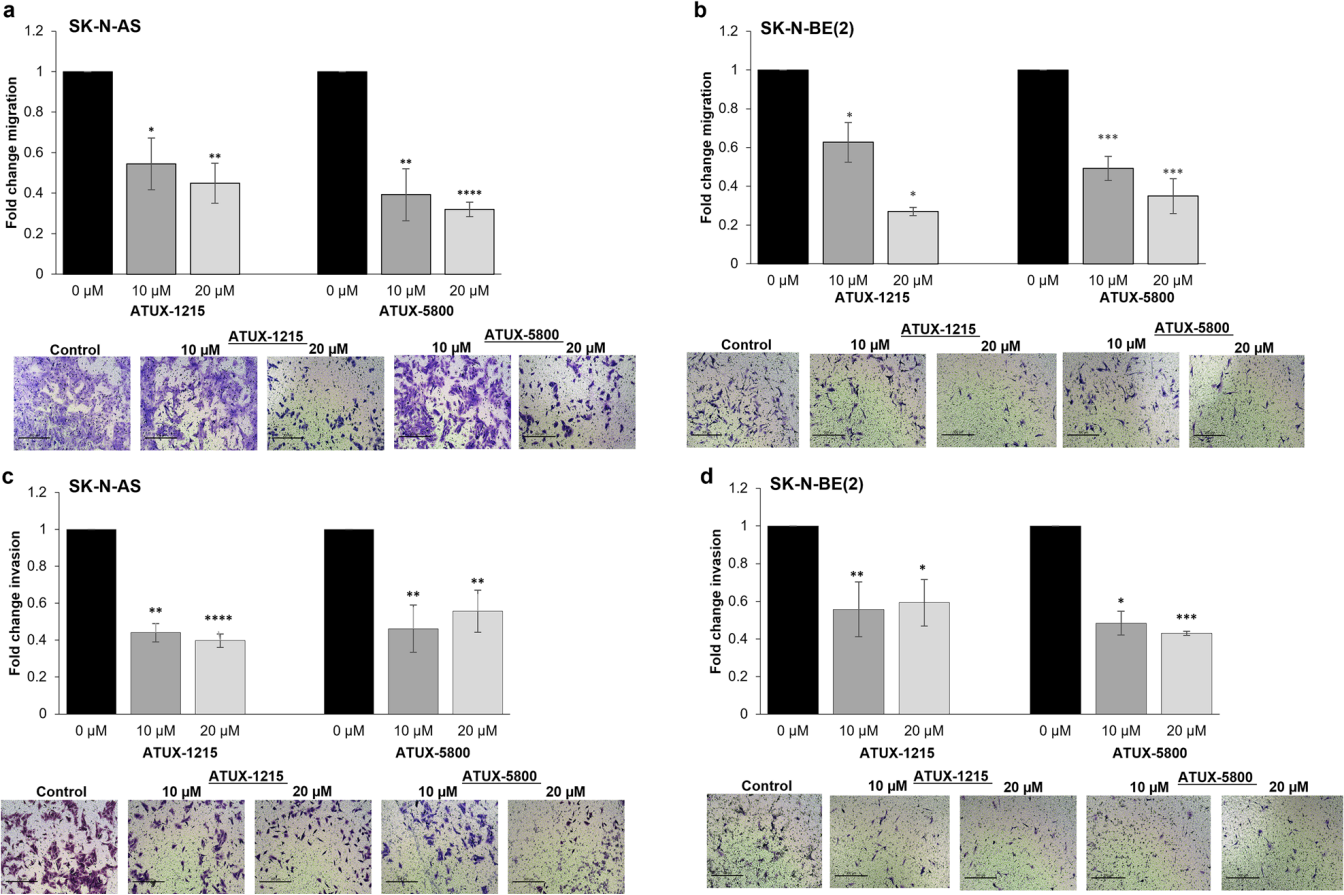
ATUX-1215 or ATUX-5800 decreased NBL in vitro migration and invasion. **a**–**d** SK-N-AS and SK-N-BE(2) NBL cells (5 × 10^5^) were pretreated with ATUX-1215 or ATUX-5800 (0, 10, or 20 μM) for 24 hours. Transwell inserts (8 μm pores) were used for migration and invasion, with the insert bottom coated with laminin for both assays and the top of inserts coated with Matrigel for invasion. Cells were allowed to migrate or invade for 24 h. Inserts were stained with crystal violet, photographed, and the number of cells migrating or invading were quantified using ImageJ. There was a significant decrease in the number of NBL cells migrating (**a**, **b**) or invading (**c**, **d**) post treatment compared to control. Representative photomicrographs of inserts from migration (**a**, **b**, *lower panels*) and invasion (**c**, **d**, *lower panels*) plates are shown. The bar graphs represent data from at least three independent biologic replicates and data reported as mean ± SEM. Data are normalized and compared to no treatment, control (1.0). Scale bars represent 300 μm (magnification 10×). Two-tailed Student’s *t*-test was employed for statistical comparison between treatment and control groups. *p ≤ 0.05, **p ≤ 0.01, ***p ≤ 0.001, ****p ≤ 0.0001. NBL, neuroblastoma; PP2A, protein phosphatase 2A.

As metastatic disease is one hallmark of high-risk NBL, we examined the effects of PP2A activation on NBL cell motility. Transwell migration assays indicated a significant decrease in migration in both SK-N-AS (Fig. 2a) and SK-N-BE(2) cells (Fig. 2b). Cells were treated with similar doses of compounds, and invasion was evaluated. There was a significant decrease in invasion of both these cell lines following treatment (Fig. 2c, d). Representative photomicrographs of migration (Fig. 2a, b, *lower panels*) and invasion (Fig. 2c, d, *lower panels*) plates are presented below graphs. Collectively, these findings demonstrate that the PP2A activating compounds may decrease NBL metastasis by affecting both migratory and invasive ability of these cells.

### Treatment with ATUX-1215 or ATUX-5800 decreases MYCN

**Fig. 3:**
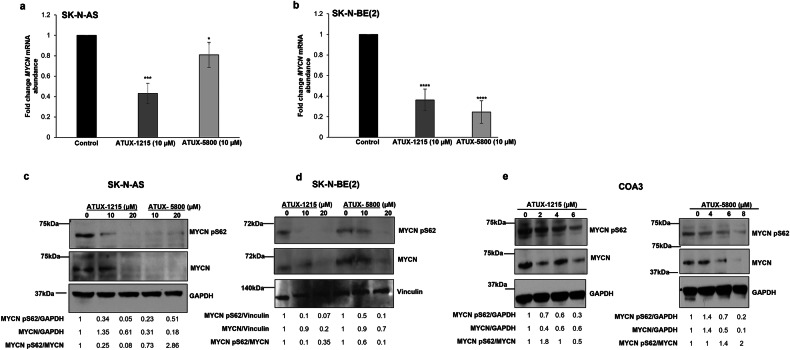
ATUX-1215 and ATUX-5800 inhibit MYCN expression. **a**–**e** SK-N-AS, SK-N-BE(2), and COA3 cells were treated with ATUX-1215 or ATUX-5800 (0–20 μM) for 24 h (SK-N-AS, SK-N-BE(2)) and for 72 hours (COA3). qRT-PCR was used to quantify *MYCN* mRNA abundance. In SK-N-AS (**a**) and SK-N-BE(2) (**b**) cells, *MYCN* mRNA abundance was significantly decreased following treatment with ATUX-1215 or ATUX-5800. **c**, **d** Immunoblotting of nuclear extracts (SK-N-AS) or whole cell lysates (SK-N-BE(2), COA3) for total MYCN and phospho-MYCN pS62 was performed. There was decreased expression of total MYCN protein and decreased phosphorylation at S62 in SK-N-AS and SK-N-BE(2) cells treated with ATUX-1215 or ATUX-5800 (**c**, **d**). COA3 cells showed decreased MYCN total protein expression after treatment with both compounds and decreased phosphorylation of S62 following treatment only with ATUX-1215 (**e**). Experiments were completed with at least three independent biologic replicates and data for qRT-PCR are reported as mean ± SEM and evaluated with two-tailed Student’s *t* test. *p ≤ 0.05, **p ≤ 0.01, ***p ≤ 0.001, ****p ≤ 0.0001. For qRT-PCR, *GAPDH* mRNA abundance was used as internal control. Vinculin or GAPDH were assayed as the loading control for immunoblotting.

We next determined whether PP2A activators would affect *MYCN* mRNA abundance and protein expression. We assessed the mRNA abundance of *MYCN* in treated and control SK-N-AS and SK-N-BE(2) cells. Treatment with either ATUX-1215 or ATUX-5800 (10 µM) significantly decreased *MYCN* mRNA level in SK-N-AS (Fig. 3a) and SK-N-BE(2) cells (Fig. 3b). In SK-N-AS, SK-N-BE(2), and COA3 PDX cells, decreased MYCN protein expression was observed following treatment with both PP2A activators (Fig. 3c–e). Decreased phosphorylation of MYCN at pS62, the site that confers stability, was observed following treatment with both PP2A activators in SK-N-AS and SK-N-BE(2) cells (Fig. 3c, d), but in the COA3 cells this effect was noted only with ATUX-1215 at the highest concentration (Fig. 3e). The effect on MYCN protein was further corroborated in COA6 PDX cells where reduced levels of MYCN protein and its phosphorylation were seen following PP2A activator treatment (Fig. S2b). These findings indicate that PP2A activation contributes to the negative regulation of MYCN by decreasing its mRNA abundance, phosphorylation, and total protein expression.

### ATUX-1215 and ATUX-5800 affect BRD4 phosphorylation

**Fig. 4:**
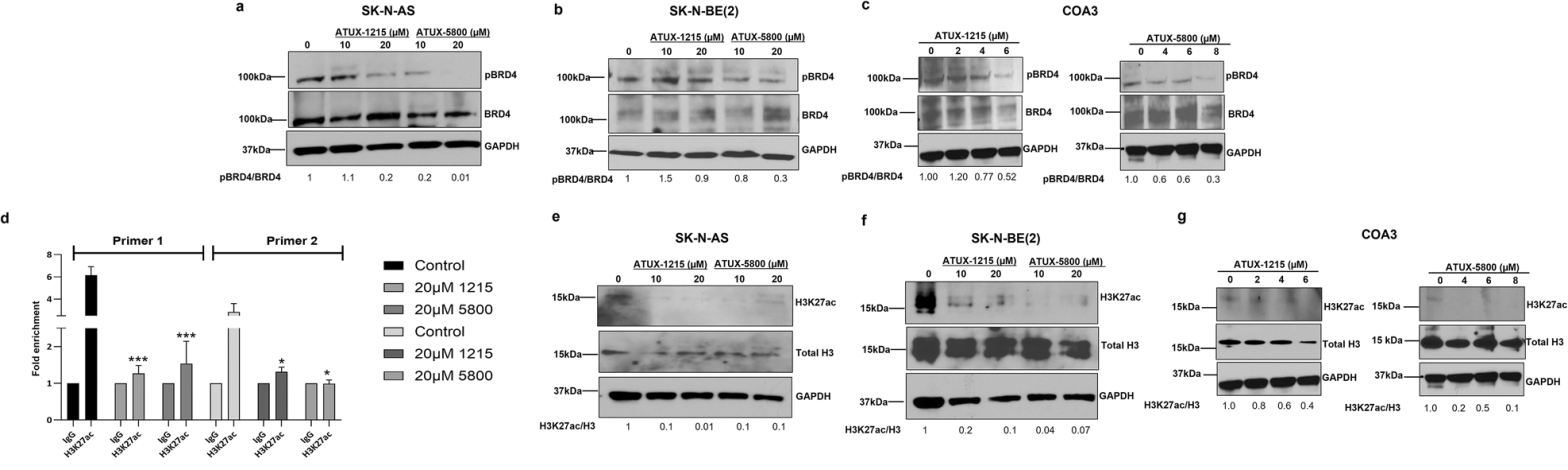
Treatment with ATUX-1215 or ATUX-5800 dephosphorylates BRD4 and reduces H3K27 acetylation. **a**, **b**, **e**, **f** SK-N-AS and SK-N-BE(2) (1 × 10^6^ cells, 24 h), and **c**, **g** COA3 PDX cells (2 × 10^6^ cells, 72 h) were treated with ATUX-1215 or ATUX-5800 (SK-N-AS and SK-N-BE(2), 0-20 μM; COA3, 0-8 μM) and immunoblotting completed for protein expression and phosphorylation of BRD4 and acetylation at H3K27. ATUX-1215 or ATUX-5800 treatment led to decreased phosphorylation of BRD4 in both cell lines (**a**, **b**) and PDX cells (**c**). **d** SK-N-BE(2) cells were treated with ATUX-1215 or ATUX-5800 (0, 20 μM) for 24 h and processed for ChIP-qPCR. ChIP-qPCR in SK-N-BE(2) control cells show enhanced H3K27ac enrichment at *MYCN* promoter (Primer 1 and 2) compared to IgG control, while H3K27ac was significantly decreased compared to control with ATUX-1215 or ATUX-5800 treatment. Immunoblotting demonstrates that H3K27 acetylation in NBL cells (**e**, **f**) or PDX cells (**g**) was reduced after treatment. GAPDH or total histone H3 served as the loading control. Data for ChIP-qPCR are reported as mean fold change enrichment to their respective IgG (1.0) ± SEM from at least three independent biological replicates. Data compared to control (no treatment) using two-tailed Student’s *t* test. *p ≤ 0.05, ***p ≤ 0.001. pBRD4, BRD4 phosphorylation at serine 492/494; H3K27ac, Histone 3 acetylation at lysine 27; 1215, ATUX-1215; 5800, ATUX-5800.

We next investigated whether BRD4 protein was affected by PP2A activation. Immunoblotting demonstrated that PP2A activation led to decreased phosphorylation of BRD4 in both NBL cell lines and the PDX cells (Figs. 4a–c, S2c). These findings motivated us to investigate the relation of BRD4 to NBL patient survival. We utilized the KOCAK neuroblastoma cohort (accession GSE45547) [31] using the R2 platform to generate Kaplan-Meier curves to answer this question. There was a negative correlation between *BRD4* abundance and event-free or overall survival in *MYCN* non-amplified (Fig. S3a, *left and right panels, respectively*) and in *MYCN* amplified patient samples (Fig. S3b, *left and right panels, respectively*).

### PP2A reactivation leads to reduced H3K27ac enrichment at *MYCN* promoter

We hypothesized that PP2A reactivation would affect histone acetylation (H3Kac), making chromatin compact and inaccessible for binding for other transcription factors or regulators, thereby inhibiting transcription of *MYCN*. We first asked if PP2A activation affects acetylation of histone 3 at lysine 27 (H3K27ac) at the *MYCN* promoter region. Our investigations thus far included both SK-N-AS and SK-N-BE(2) cells to identify PP2A-reactivation based phenotypic changes. Results were similar in both cell lines, irrespective of *MYCN* amplification, so we focused our next studies on *MYCN* amplified cells, SK-N-BE(2), since *MYCN* amplified tumors are clinically the most challenging to treat.

We performed chromatin immunoprecipitation coupled with quantitative PCR (ChIP-qPCR) to assess the enrichment of H3K27ac on the *MYCN* promoter following treatment with ATUX-1215 or ATUX-5800 (0, 10, 20 µM). Reduction in H3K27ac abundance on the *MYCN* promoter was seen with ATUX-1215 or ATUX-5800 (20 µM, Fig. 4d) compared to control. Similar ChIP qPCR results were obtained with doses of 10 µM (Fig. S4). Hence, reactivation of PP2A is associated with a decrease in H3K27ac on the promoter region for *MYCN*, providing a potential mechanism for the decrease in *MYCN* transcription seen with PP2A activator treatment.

Next, we examined whether PP2A activators affected the acetylation status of histone H3 to corroborate our ChIP findings. ATUX-1215 and ATUX-5800 treatment resulted in mixed histone H3 acetylation expression of H3K9ac, H3K27ac, and H3K122ac, all marks associated with open chromatin and active transcription in both SK-N-AS and SK-N-BE(2) (Fig. 4e, f; Fig. S5a, b) and COA3 (Fig. 4g) COA6 (Figs. S2d and S5c) cells. H3K27 acetylation was the most consistently reduced epigenetic mark upon PP2A activation across all NBL cells (Fig. 4e–g). Acetylation of H3K9 and H3K122 was more variable (Fig. S5a–c). We therefore conclude that PP2A activators decreased pBRD4 and reduced histone H3K27 acetylation which may contribute to decreased *MYCN* transcription due to repression of *MYCN* transcription machinery.

### ATUX-1215 and ATUX-5800 treatment dephosphorylates RNA pol II CTD

**Fig. 5:**
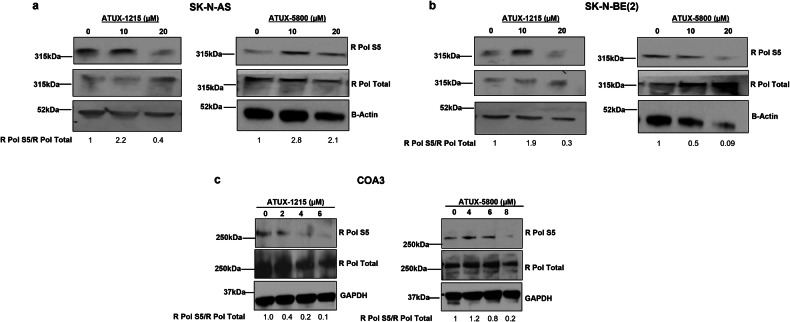
ATUX-1215 or ATUX-5800 treatment alters phosphorylation state of RNA Polymerase II C terminal domain (R Pol II CTD). **a**–**c** SK-N-AS, SK-N-BE(2), and COA3 (1 × 10^6^ cells) were treated with increasing concentrations of ATUX-1215 or ATUX-5800 (SK-N-AS or SK-N-BE(2), 0–20 μM, 24 h; COA3, 0–8 μM, 72 h) and expression of total R Pol and phosphorylation at S5 was detected using immunoblotting. In SK-N-AS cells, decreased phosphorylation of R Pol S5 was noted only at the 20 μM concentration of ATUX-1215 (**a**, *left panel*). In SK-N-BE(2) cells, R Pol phosphorylation at S5 was decreased following 20 μM ATUX-1215 treatment (**b**, *left panels*), and ATUX-5800 reduced phosphorylation of R Pol at S5 at both concentrations (**b**, *right panels*). Phosphorylation of R Pol S5 was decreased by both compounds in the COA3 cells (**c**). β-actin or GAPDH served as loading controls.

Since previous studies found that PP2A activation affects RNA pol II [14], we determined the effects of ATUX-1215 and ATUX-5800 on RNA pol II. In SK-N-AS cells, phosphorylation of R Pol S5 was decreased by 20 µM ATUX-1215 (Fig. 5a, *left panel*) but phosphorylation of these proteins was increased with a decrease in the total protein by ATUX-5800 (Fig. 5a, *right panel*). In the SK-N-BE(2) cells, both compounds decreased phosphorylation of R Pol S5 (Fig. 5b). Parallel investigation in PDX cells showed both PP2A activating compounds reduced phosphorylation of R Pol S5 (Figs. 5c and S2e).

For R Pol S2 phosphorylation, variable results were noted for both ATUX-1215 or ATUX-5800 treated NBL cell lines, SK-N-AS (Fig. S6a) and SK-N-BE(2) (Fig. S6b). In COA6 PDX cells, both PP2A activators led to a decrease in R Pol S2 phosphorylation (Fig. S6c). Hence, we conclude these PP2A activators dephosphorylate R Pol CTD II at serine residues, resulting in hypophosphorylated R Pol II, potentially contributing to *MYCN* repression at the transcriptional level.

### PP2A reactivation inhibits NBL tumor growth in vivo

**Fig. 6:**
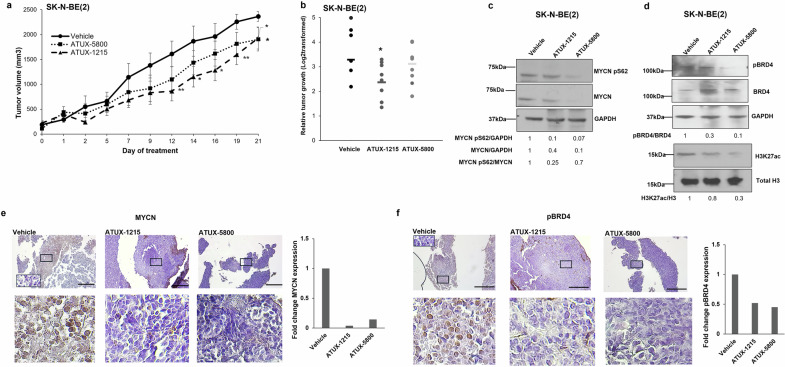
ATUX-1215 and ATUX-5800 decreased SK-N-BE(2) tumor growth in vivo by decreasing MYCN, BRD4 phosphorylation, and H3K27 acetylation. **a**–**f** SK-N-BE(2) cells (1.5 × 10^6^) were injected into the right flank of female athymic nude mice. Once tumors reached 100 mm^3^, animals were randomized into three groups: Vehicle (*n* = 7), ATUX-1215 (*n* = 8, 75 mg/d), and ATUX-5800 (*n* = 9, 75 mg/d), and treated via oral gavage twice daily for 21 days. Animals treated with ATUX-1215 showed decreased tumor volumes compared to controls beginning at 12 days (**a**, *dashed line, triangles*). Treatment with ATUX-5800 resulted in decreased tumor volumes at the end of the experiment (**a**, *dotted line, squares*). When examining change in tumor growth, ATUX- 1215 had a statistically significant decrease compared to vehicle (**b**). **c**–**f** Tumor tissues from these animals were used to prepare whole cell lysates for immunoblotting and tissue slides for immunohistochemical (IHC) staining. MYCN protein expression and its phosphorylation (MYCN pS62) were decreased in tumor lysates from animals treated with ATUX-1215 and ATUX-5800 with respect to vehicle-treated controls (**c**). Decreased BRD4 phosphorylation (pBRD4) and reduction of H3K27ac was present in tumor lysates from both ATUX-1215 and ATUX-5800 treated groups compared to vehicle treated control animals (**d**). IHC staining for MYCN shows fewer cells staining positive in tumors from animals treated with ATUX-1215 or ATUX-5800 (**e**, *brown stain*). Negative control rabbit IgG staining was included (*inset, lower left corner of upper left panel*). Tumors were stained for phosphorylated BRD4 (pBRD4). Staining for pBRD4 was reduced in tumors from animals treated with ATUX-1215 or ATUX-5800 compared to vehicle controls (**f**, *brown stain*). Negative control rabbit IgG staining was included (**f**, *inset, upper left corner of upper left panel*) with each IHC run. Magnification of 10× for top panels and 40× for bottom panels. Bottom panels represent the area outlined by the black boxes in the upper photomicrographs. All scale bars represent 1 mm. Data are presented as mean tumor volume ± SEM. Statistical comparison was completed with two tailed Student’s *t* test comparing vehicle treated controls to ATUX-1215 or ATUX-5800 treated animals. *p = 0.05, **p = 0.01.

Based on results from in vitro experiments, and the apparent multipronged effect of PP2A activation on MYCN through effects on transcription, post-translational modification, and protein stability, we next investigated the therapeutic application of ATUX-1215 and ATUX-5800 in vivo. Tumors were established by injecting SK-N-BE(2) NBL cells in the right flank of athymic nude mice. Data on oxidative stability, clearance rates, and previous in vivo studies were taken into consideration for dose selection, and oral dosing twice daily (bid) at 75 mg/kg was adopted [8, 27, 30, 32]. This dosing regimen was well tolerated with no significant change in animal weight over the study period (data not shown). SK-N-BE(2) tumors grew more slowly in ATUX-1215 or ATUX-5800 treated mice compared to vehicle control animals (Fig. 6a). The relative tumor growth was significantly decreased in ATUX-1215 treatment group compared to vehicle treated animals (Fig. 6b). Comparing the anti-tumor response of the two compounds suggests that ATUX-1215 had a more marked effect on tumor growth than ATUX-5800. Tumor bearing mice treated with ATUX-1215 showed an increased amount of time for tumors to reach 2500 mm^3^ (median time = 20 days) compared to vehicle treated animals (median time = 14 days) (Fig. S7a) although it did not reach statistical significance.

We performed immunoblotting on the tumor tissues harvested from the animal experiment to assess the expression of MYCN, BRD4, and their respective phosphorylation along with H3K27 acetylation. MYCN phosphorylation and expression was decreased in tumor lysates from animals treated with ATUX-1215 or ATUX-5800 (Fig. 6c). Treatment with ATUX-1215 or ATUX-5800 led to decreased phosphorylation of BRD4 (Fig. 6d, *upper panels*) and decreased acetylation of H3K27 (Fig. 6d, *lower panels*). These results suggest that decreased tumor growth may be due to MYCN inhibition potentially *via* PP2A regulated MYCN dephosphorylation and decreased transcription.

IHC staining of the tumors further substantiated the in vitro and in vivo results. MYCN staining was decreased in the tumors treated with ATUX-1215 or ATUX-5800 compared to tumors from vehicle treated animals (Fig. 6e, *brown staining*). There was decreased pBRD4 staining in the tissues of ATUX-1215 or ATUX-5800 treated animals compared to those treated with vehicle (Fig. 6f, *brown staining*). Tissues were stained for Ki67, a marker of cell proliferation. There were fewer cells staining for Ki67 in the ATUX-1215 and ATUX-5800 treated tumors compared to vehicle controls (Fig. S7b, *brown staining*). These data indicate that PP2A activators, ATUX-1215 and ATUX-5800, decrease tumor growth in vivo, likely through decreased MYCN expression.

**Fig. 7:**
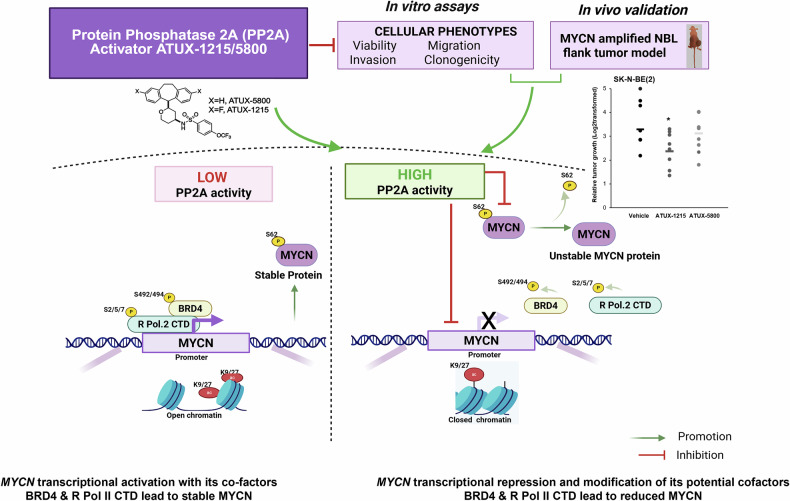
Cartoon representing findings. PP2A activation may decrease MYCN-driven NBL through several pathways including decreased acetylation of H3K27 at MYCN promoter, decreased phosphorylation of BRD4, decreased phosphorylation of R Pol II CTD, and the post-translational modifications of MYCN protein. PP2A, protein phosphatase 2A; NBL, neuroblastoma; BRD4, bromodomain-containing 4, a BET protein; R Pol II CTD, RNA polymerase II C terminal domain; K9/K27ac, acetylation at lysine 9 or 27. S, serine; T, threonine. Figure created with BioRender (BioRender. Beierle, E. (2025) https://BioRender.com/u96f056).

Figure 7 is a brief cartoon of the relevant findings of these studies.

## Discussion

This study establishes the clinical relevance of novel PP2A activators in NBL by identifying the potential mechanisms of MYCN inhibition including transcription, post-translation modification, and protein stability through pharmacologic PP2A reactivation. We demonstrated that treatment with PP2A activators resulted in dephosphorylation of the BRD4 and RNA pol II CTD proteins, decreased H3K27 acetylation at the *MYCN* promoter, and alterations in MYCN protein expression and phosphorylation.

Transcription factors, such as MYCN, are known PP2A substrates [33] which provided an impetus for us to search for a link between PP2A and MYCN driven or addicted NBL [4, 8, 12]. We noted that treatment with ATUX-1215 or ATUX-5800 significantly decreased *MYCN* mRNA abundance in NBL cells but, *MYCN* amplified SK-N-BE(2) treated cells tended to have a greater fold inhibition in *MYCN* mRNA abundance than the non-amplified SK-N-AS cells. This finding may reflect the reliance of MYCN addicted tumors on low PP2A activity for survival. Other studies support this supposition. Reduction of the PP2A catalytic subunit by knock-down decreased NBL growth, but complete ablation by knock-out was not tolerated by the cells [9]. The investigators of that study surmised that their findings implied that NBL cells may be addicted to PP2A, because of the resultant protumorigenic MYCN activation. They felt that there were opposing functions of *PP2A* gene expression that may be dose dependent; first, as an essential survival gene when expressed in low amounts, and second, as a tumor suppressor when expressed at high levels, and that tumors required a low amount of PP2A to support other oncogenes such as *MYCN* [9].

Other investigators have found that the anti-tumor effects of small molecule-based PP2A activators, when tested in models of MYC-driven cancers such as Burkitt lymphoma, KRAS-driven non–small cell lung cancer, or triple-negative breast cancer, were related to the ability of PP2A to decrease the *MYC* oncogene on the transcriptional and protein stability levels [12]. These studies provide support for our findings that the tumor suppressor function seen with PP2A activation is strongly related to its effects on the *MYCN* oncogene.

In addition to identifying PP2A as a negative regulator of *MYCN* gene expression, we also saw PTMs responsible for reduced MYCN protein expression through dephosphorylation of Ser 62 (MYCN S62). S62 phosphorylation confers MYCN protein stability and functional activation [34, 35]. We noted decreased S62 phosphorylation and decreased total MYCN protein expression following ATXU-1215 and ATUX-5800 treatment, suggesting that changes in phosphorylation may be one of the mechanisms contributing to decreased MYCN protein expression. MYCN stimulates its own transcription through a positive regulatory loop that leads to its high gene expression [36, 37]. Hence, its reduced protein expression and stability is a crucial factor behind its own transcription repression.

A series of site-specific phosphorylation and dephosphorylation events determines the binding specificity of the R Pol II CTD with its accessory factors. The best-studied events occur at serine 5 phosphorylation (pS5) which is predominant at initiation, and serine 2 phosphorylation (pS2) which is increased during elongation [38]. We found that PP2A activators, ATUX-1215 and ATUX-5800, led to reduced R Pol II CTD S5 and S2 phosphorylation, implicating reduced transcriptional initiation as well as elongation, respectively, as responsible mechanisms. Other investigators have seen similar effects on R Pol II CTD with other PP2A activators. Their findings suggested that binding of these molecules to the PP2A holoenzyme enabled the formation of an integrator protein-PP2A (Int-PP2A) complex [14, 39, 40]. Specifically, PP2A binding with Integrator complex S6/8 dephosphorylates residues within the R Pol II CTD preventing productive elongation and playing a crucial role in transcriptional repression [14, 41]. Also, the interaction with Integrator-RNAP II provides a rationale for employing PP2A activators in transcriptionally addicted cancers, such as NBL [42, 43].

In the current study, we observed some increased readout of phosphorylated serine residues on R Pol II. This finding may be attributed to various factors such as phosphatases, dedicated kinases, chromatin modifications, and signal transduction pathways that affect the transcription cycle by determining the pattern of CTD phosphorylation, a highly dynamic process [44]. High S5 phosphorylation is also indicative of R Pol II involvement in transcribing the first few hundred nucleotides of genes [45], while increased levels of S2 phosphorylation come into play when R Pol II moves away from the promoter. The increase in S5 or S2 phosphorylation was evident at mostly low doses of the PP2A activators, possibly as a cellular compensatory mechanism towards loss of *MYCN* oncogenic signaling. Phosphorylation declined at higher doses (20 µM in cell lines), possibly reflecting the inability of the cells to compensate for increased PP2A activation, leading to their dephosphorylated state and decreased MYCN transcription.

PP2A regulates the activity of BRD4 [17], a transcriptional and epigenetic regulator that accumulates at euchromatic regions promoting active transcription of key protooncogenes including *MYCN* [18, 20]. BRD4 protein associates with chromatin via interactions with diverse histone acetylation marks and of these, BRD4 bound H3K27ac sites have been associated with high gene activity including *MYCN* [18]. Further, phosphorylated BRD4 recruits pTEFb to the R Pol complex which alleviates premature pausing of elongation. Thus, decreased BRD4 phosphorylation upon PP2A reactivation seen in our results possibly contributed to *MYCN* epigenetic repression due to compaction of the transcriptional machinery and disruption of transcriptional elongation. These findings have translational relevance, as we found an inverse clinical correlation between *BRD4* gene expression and event free and overall survival in NBL.

In the current study, treatment with either ATUX-1215 or ATUX-5800 decreased cell viability in NBL cell lines and in human NBL PDX cells. The COA6, COA3 and COA129 PDX cells appeared to be non-inferior to the established cell lines suggesting that these activators may be clinically translatable as PDXs are thought to recapitulate the human condition better than established cell lines in that they maintain the genetic features and characteristics of the tumor microenvironment seen in the original tumor. Often, responses to interventions seen with established cell lines do not translate to the clinical realm. Therefore, seeing a response to treatment in these PDX cells may portend well for the positive clinical translation of these treatments. One limitation to PDX cells is that they do not grow in standard culture conditions, and they grow slowly and unpredictably in animals, thereby limiting their use for many in vitro phenotypic assays such as anchorage independent growth and motility studies, and in vivo studies.

These current studies provide pre-clinical evidence of the inhibitory effects of PP2A activation on NBL tumorigenesis by affecting *MYCN* at the mRNA and protein levels via epigenetic regulation. Our findings gain credence from earlier studies emphasizing the consequences of MYCN inactivation leading to sustained tumor regression [46, 47]. Although both ATUX-1215 and ATUX-5800 affected MYCN at the protein and gene level in vitro, we noted that ATUX-1215 was the better anti-tumor compound in vivo. We attribute this finding to prolonged exposure in vivo due to the greater oxidative stability and lower clearance seen in ATUX-1215 versus ATUX-5800 [30]. We also noted target engagement in the tumor tissues, with decreased expression of MYCN, pBRD4, and H3K27ac, strengthening the hypothesis that these are the mechanisms involved. We recognize that PP2A activation alone was not sufficient to completely abolish tumor growth in the in vivo model, but this finding is not unexpected considering the redundant survival mechanisms that solid tumors harbor.

Direct PP2A activators have shown promising substrate specificity by targeting specific PP2A regulatory complexes, potentially enhancing efficacy while diminishing potential unwanted effects. Inhibitors designed to target the endogenous inhibitors of PP2A such as SET or CIP2A have not realized this specificity, providing support for direct PP2A activators [48]. Further, direct PP2A activators provide alternatives for dual inhibition with drugs such as BRD4 inhibitors and EZH2 inhibitors that have shown some efficacy in NBL but not significant or sustained results [49–51].

To the best of our knowledge, this is the first study to provide pre-clinical evidence of an antitumor effect in NBL employing a PP2A-centric epigenetic approach to target MYCN and underscores the therapeutic usefulness of pharmacologic PP2A activation as a novel means to target what has previously been considered an undruggable target. We demonstrate effectiveness against MYCN at several levels including gene transcription and PTMs resulting in decreased phosphorylation and protein expression. We believe that PP2A activation may be helpful as a therapeutic component to NBL treatment, especially for high-risk, difficult to treat tumors.

## Supplementary information

Uncropped blot

Supplementary figure legends

Figure S1

Figure S2

Figure S3

Figure S4

Figure S5

Figure S6

Figure S7
