# Supplementary material for: PP2A activation targets MYCN in neuroblastoma
Source: Cell Death Dis. 2026 Jan 15;17(1):42. doi: 10.1038/s41419-025-08253-0 (PMC12808165; doi:10.1038/s41419-025-08253-0)

Figure 3.c

SK-N-AS

	ATUX-1215 (µM)			ATUX- 5800 (µM)	
	0	10	20	10	20

MYCN p.S62 **MYCN**

GAPDH

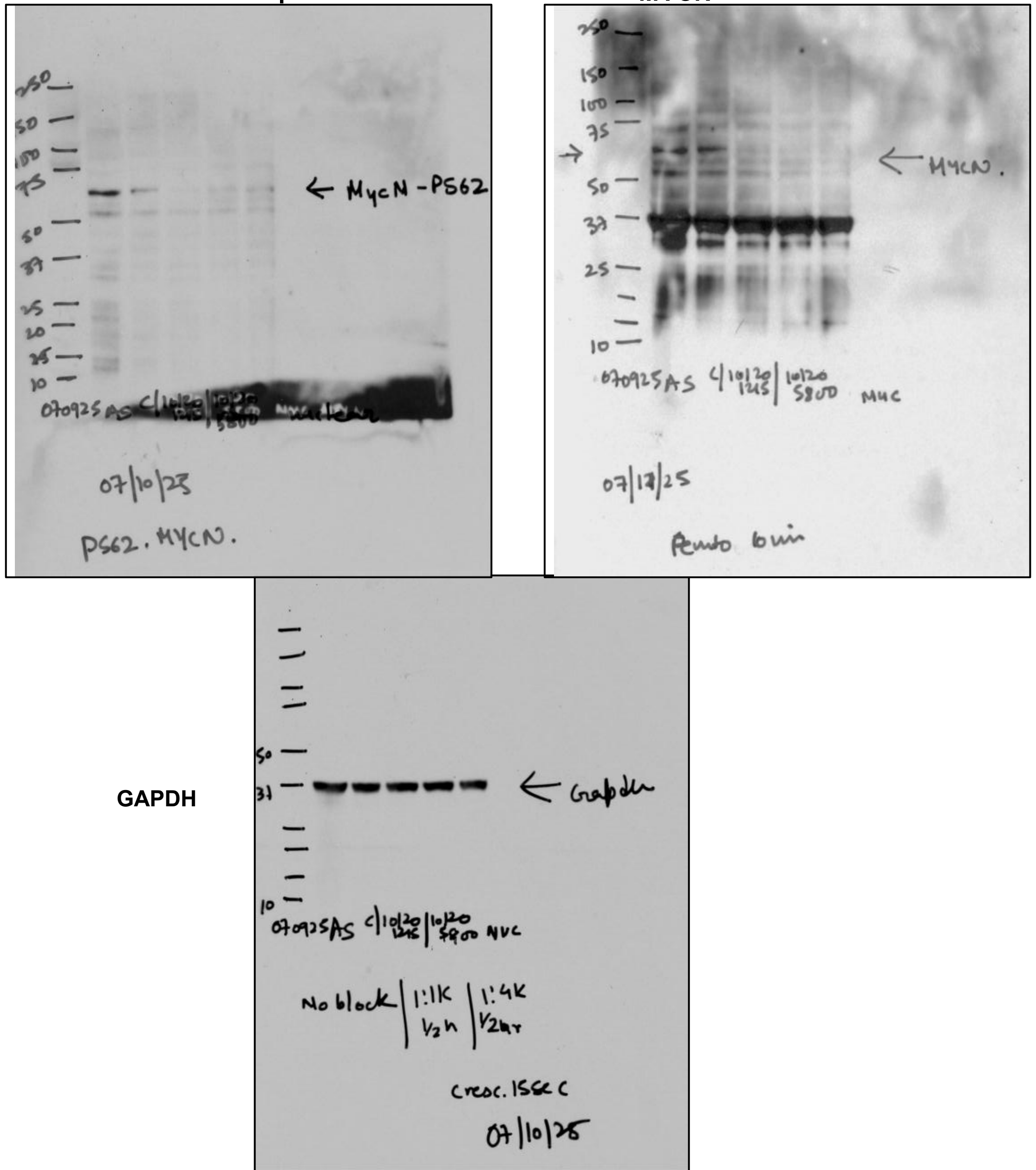

Figure 3.d

SK-N-BE(2)

ATUX-1215 | ATUX- 5800

0 10 20 | 0 10 20 (µM)

MYCN p.S62 | MYCN

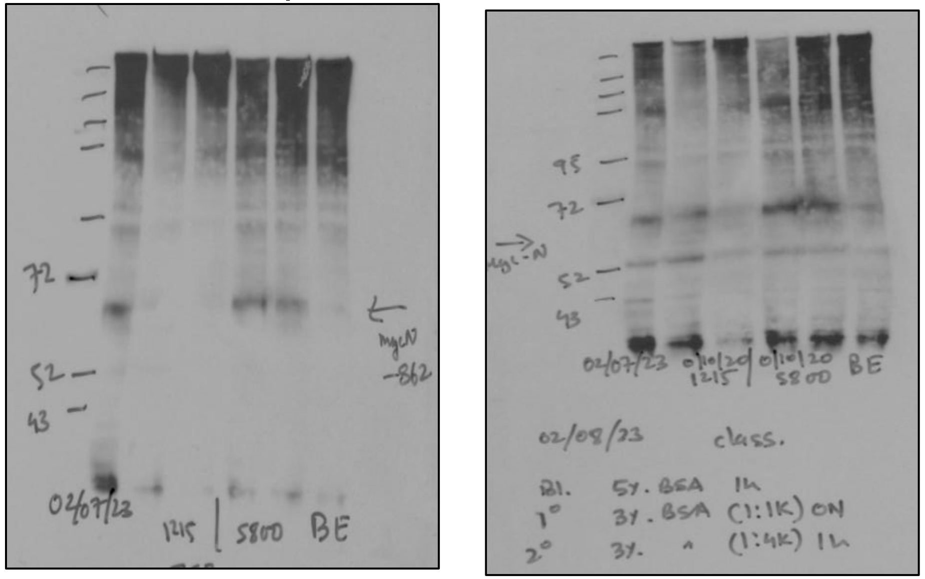

Vinculin

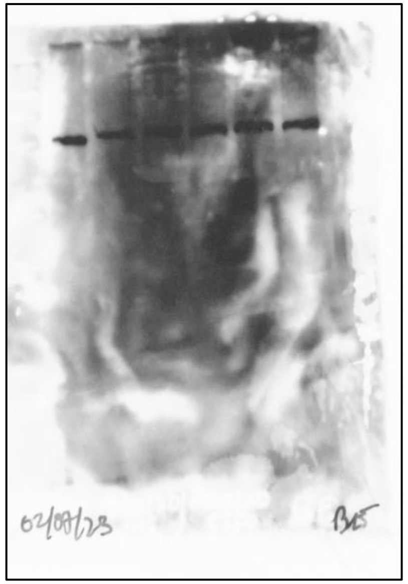

Figure 3.e

COA3

ATUX-1215

0 2 4 6 (µM)

MYCN pS62 | MYCN | GAPDH

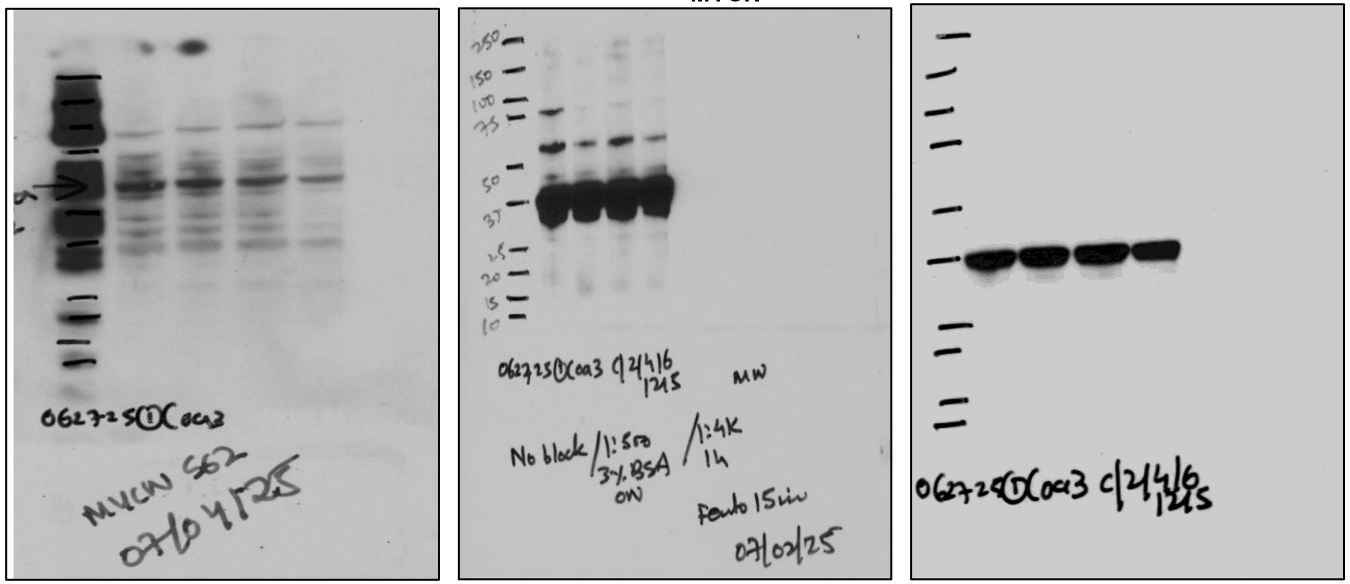

ATUX- 5800

0 4 6 8 (µM)

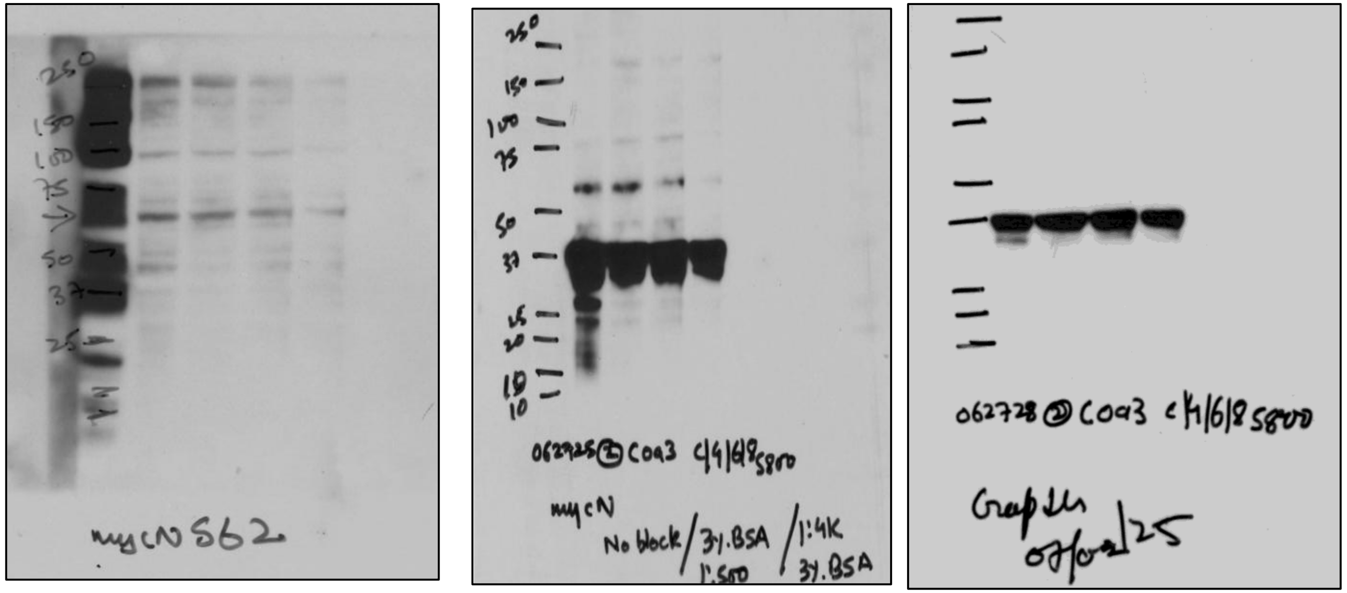

Figure 4a **SK-N-AS**

	ATUX-1215 (µM)		ATUX-5800 (µM)	
0	10	20	10	20

pBRD4 BRD4 GAPDH

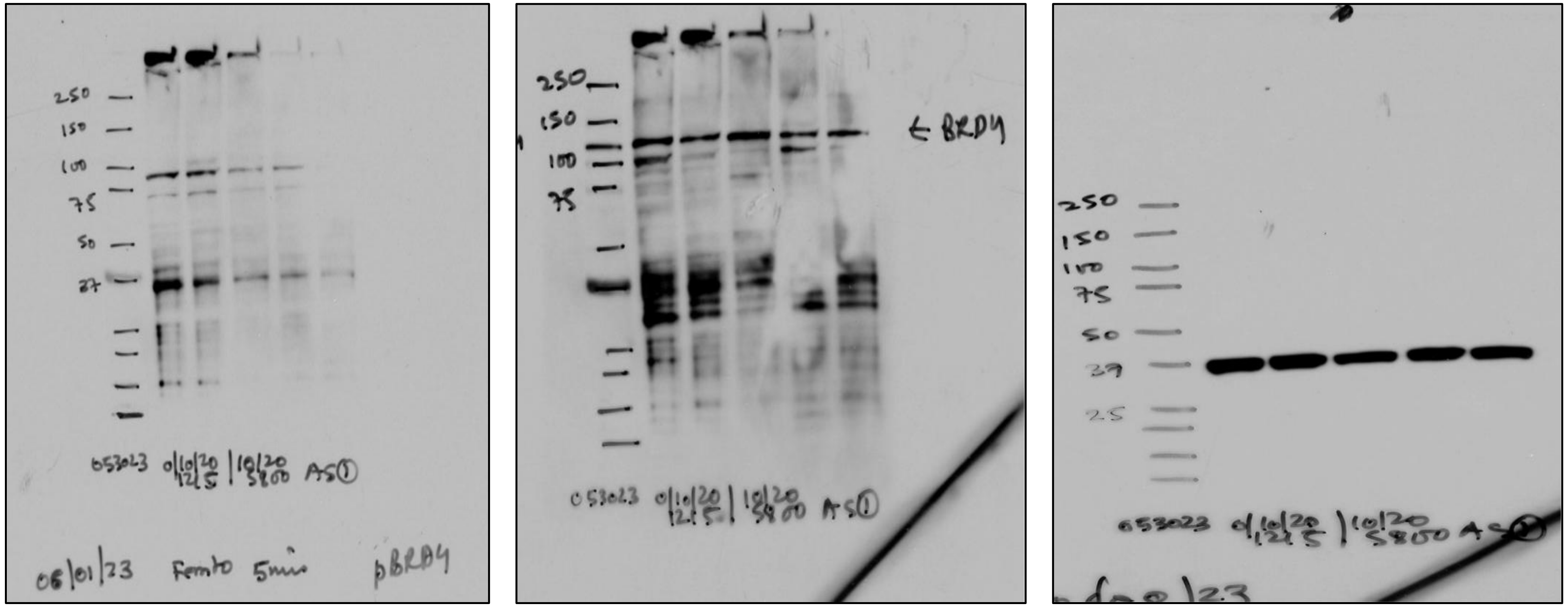

Figure 4b **SK-N-BE(2)**

	ATUX-1215 (µM)		ATUX-5800 (µM)	
0	10	20	10	20

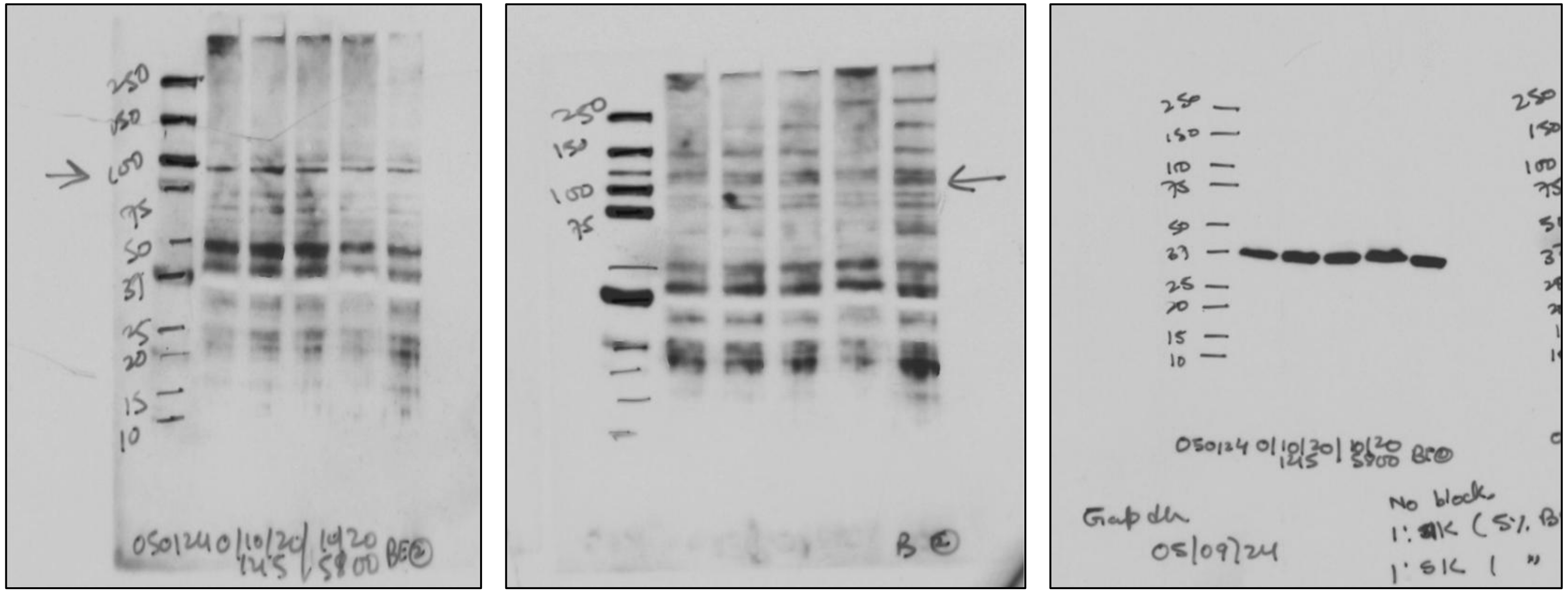

Figure 4c COA3

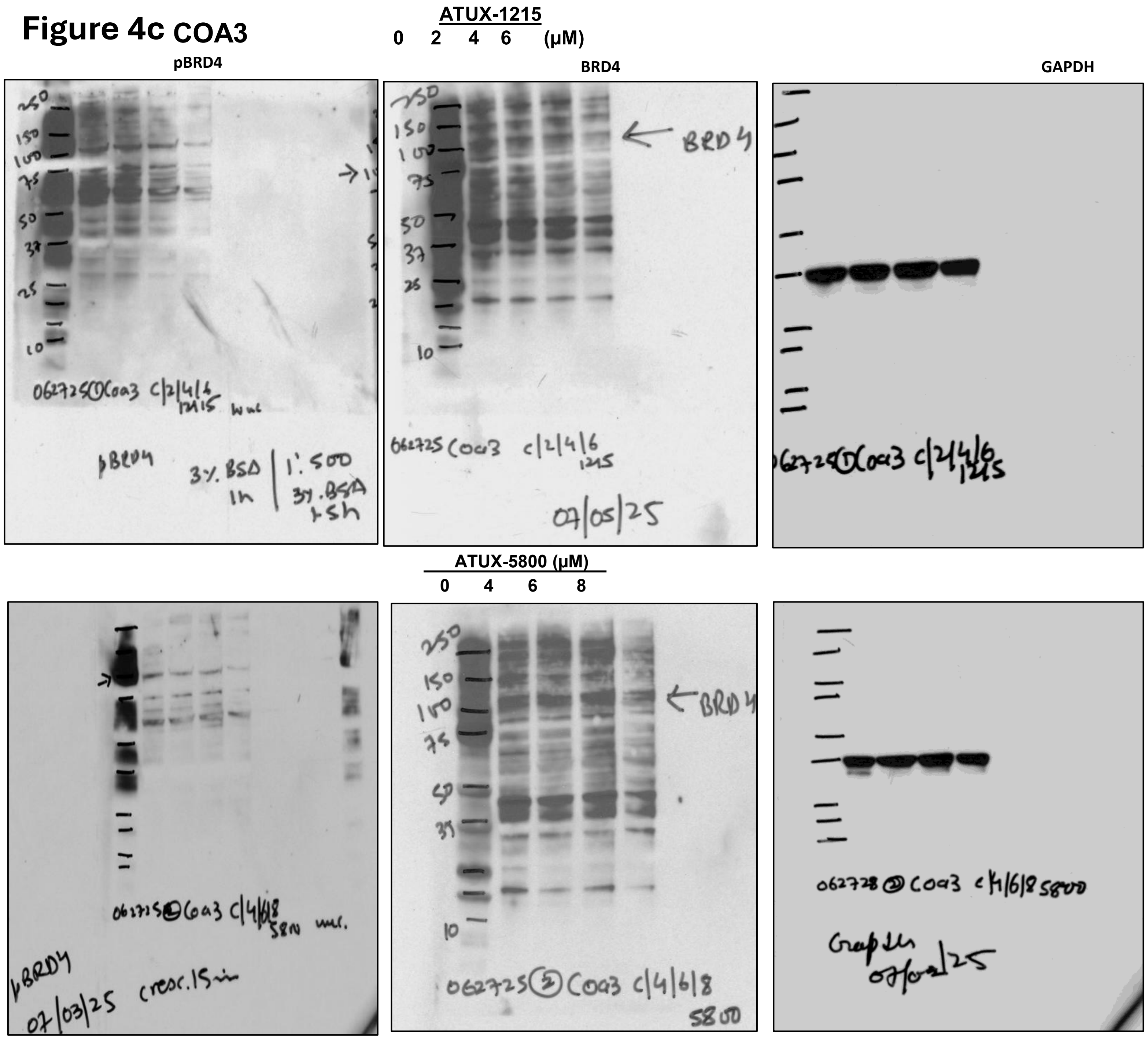

Figure 4e SK-N-AS

ATUX-1215 (μM) ATUX-5800 (μM)

0 10 20 10 20

H3K27ac Total H3 GAPDH

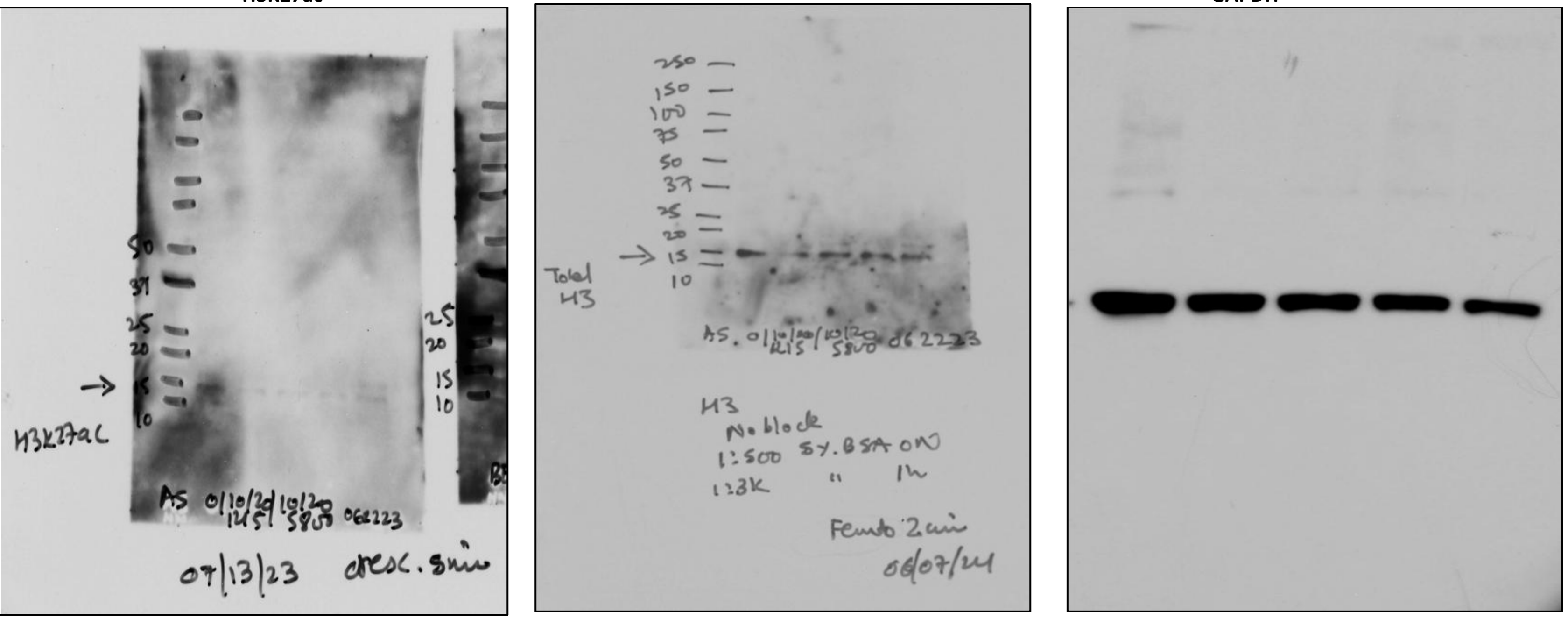

Figure 4f

ATUX-1215 (μM) ATUX-5800 (μM)

0 10 20 10 20

H3K27ac Total H3 GAPDH

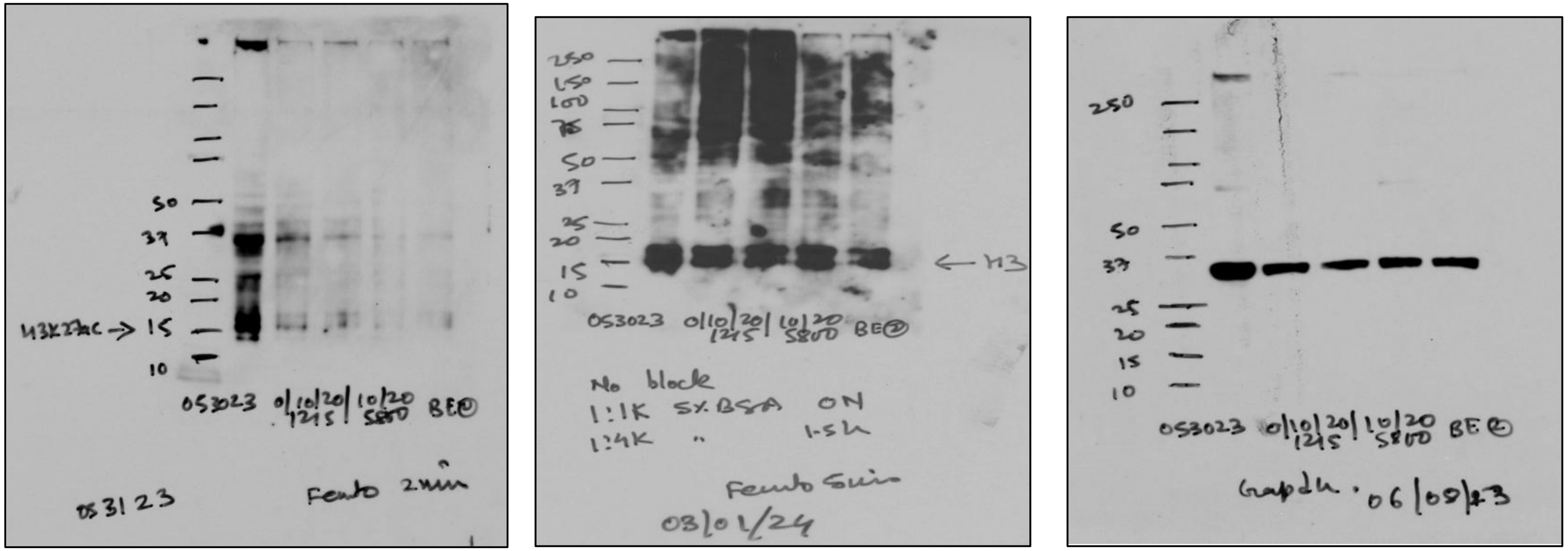

Figure 4g COA3

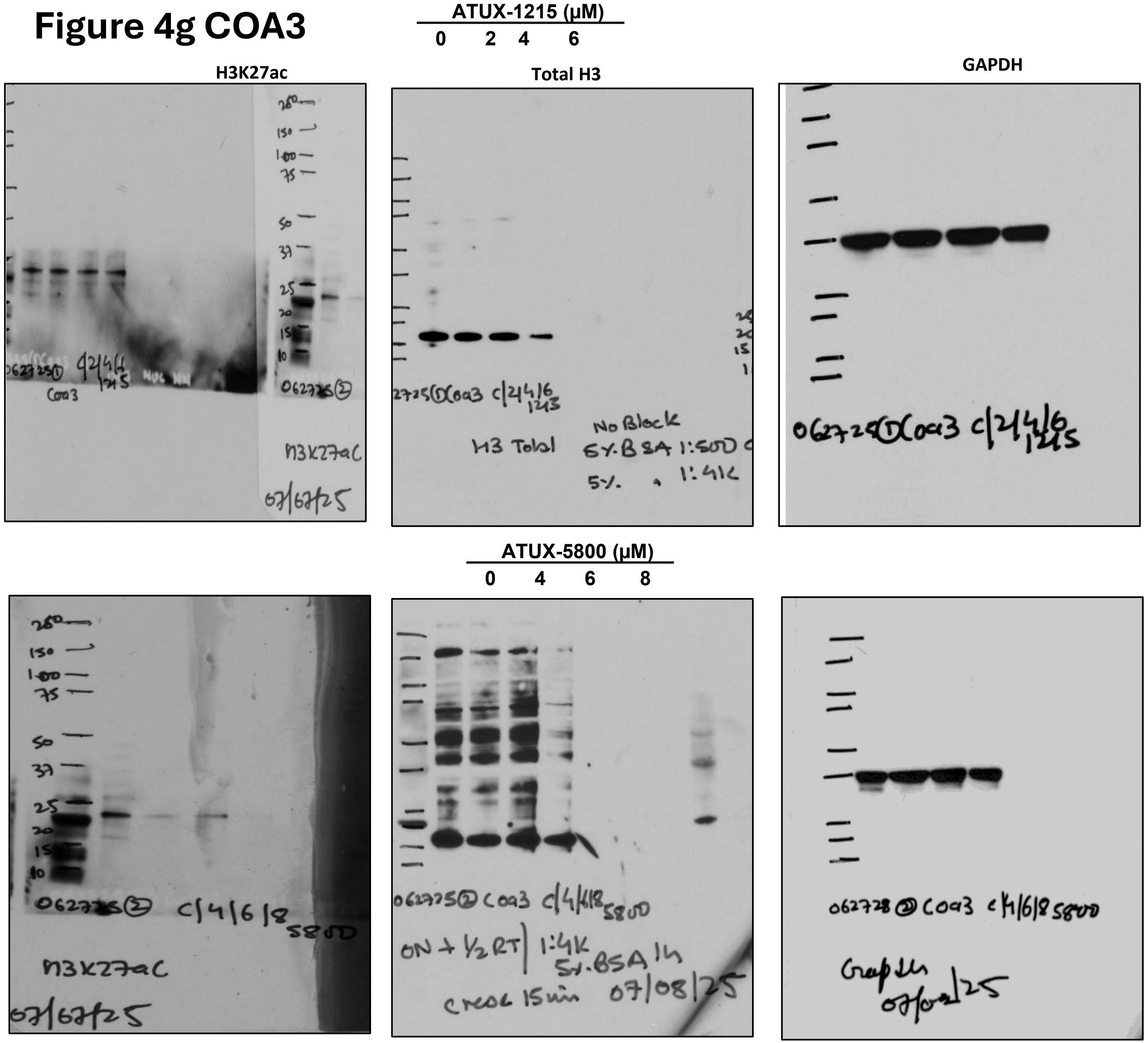

Figure 5a

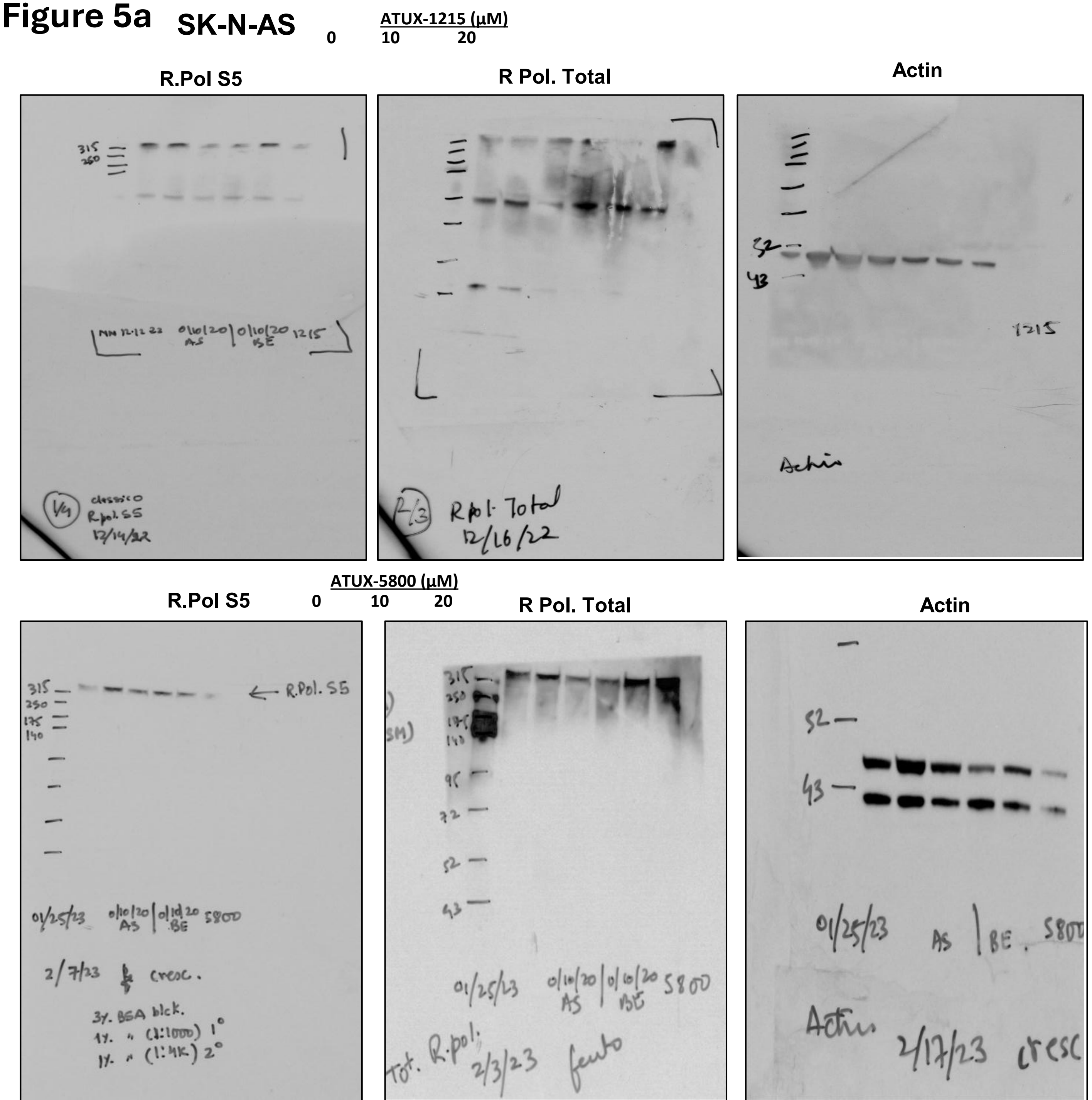
SK-N-AS
ATUX-1215 (μM)
0
10
20
R.Pol S5
R Pol. Total
Actin
ATUX-5800 (μM)
0
10
20
R.Pol S5
R Pol. Total
Actin

Figure 5b **SK-N-BE(2)**

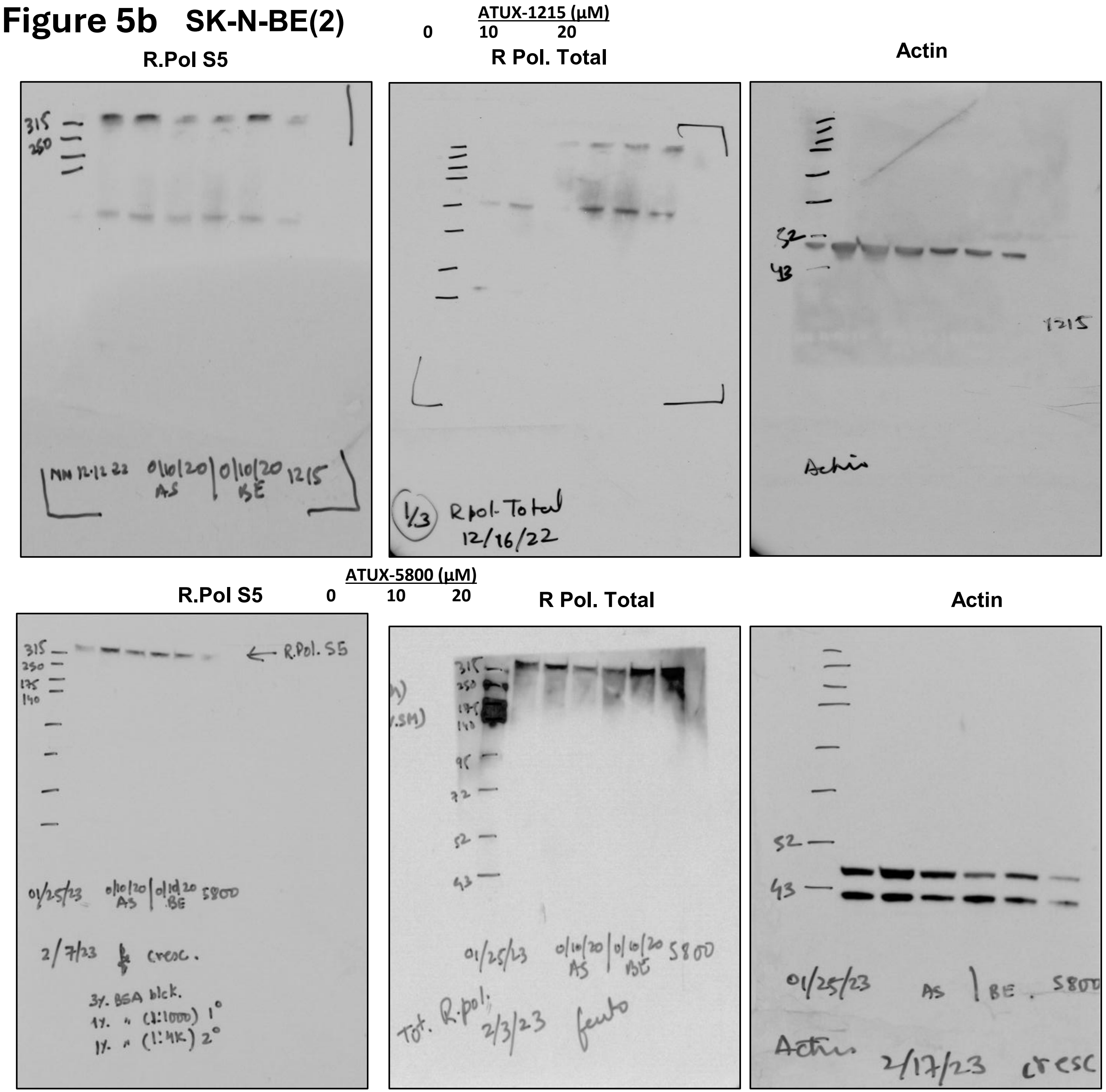

Figure 5c COA3

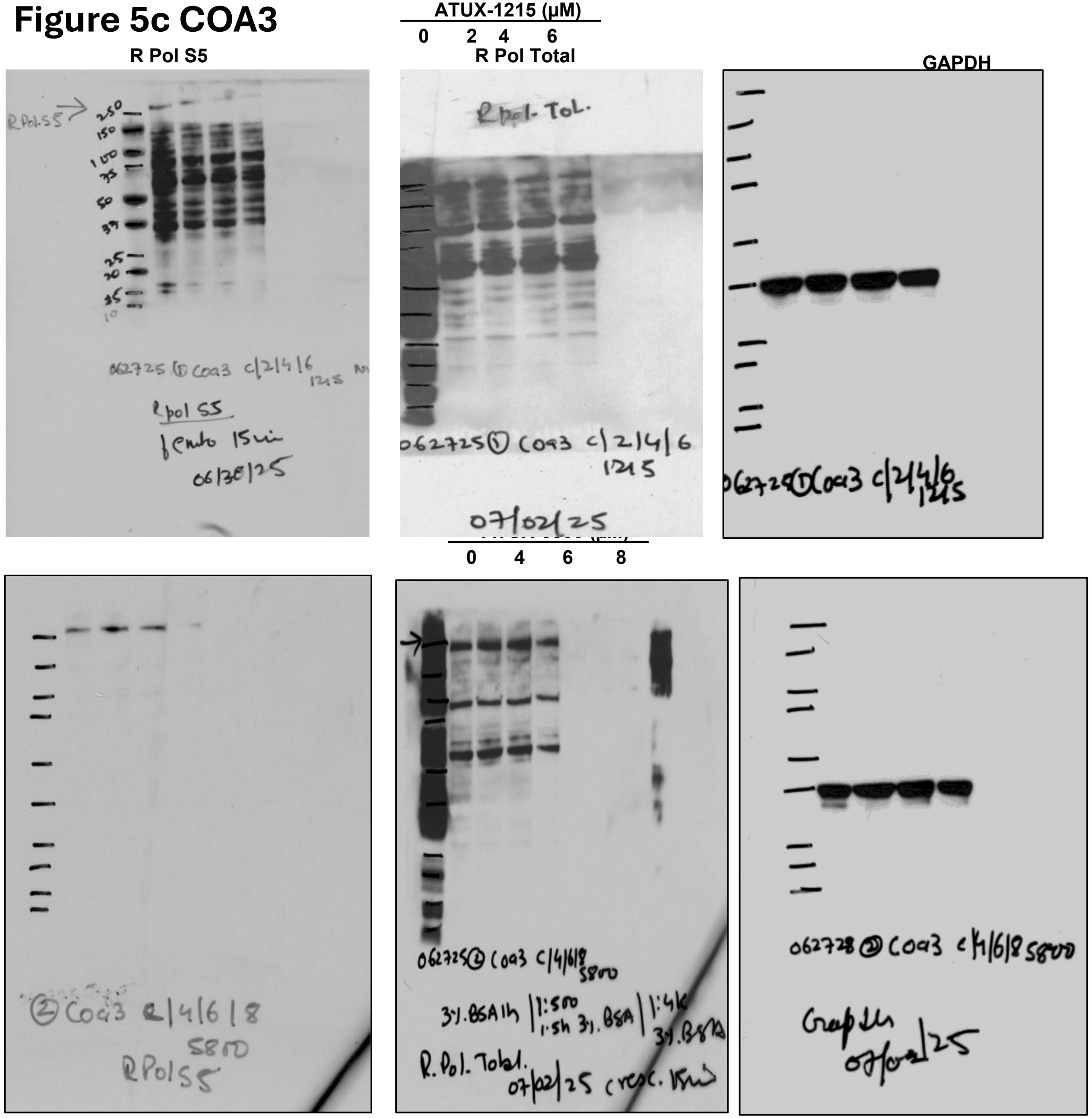

Figure 6c **vehicle/1215/5800**

MYCN p.S62

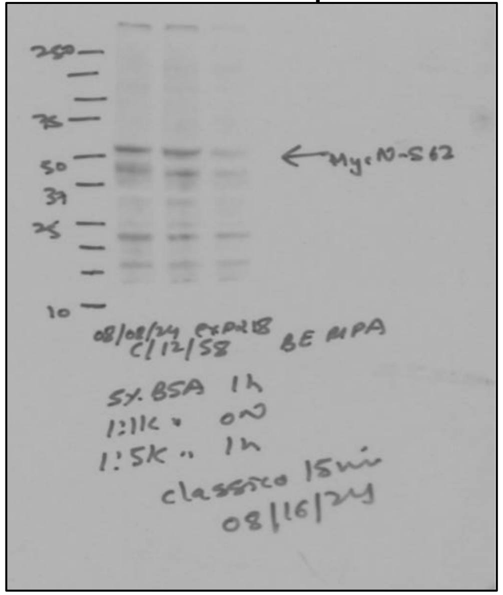

MYCN

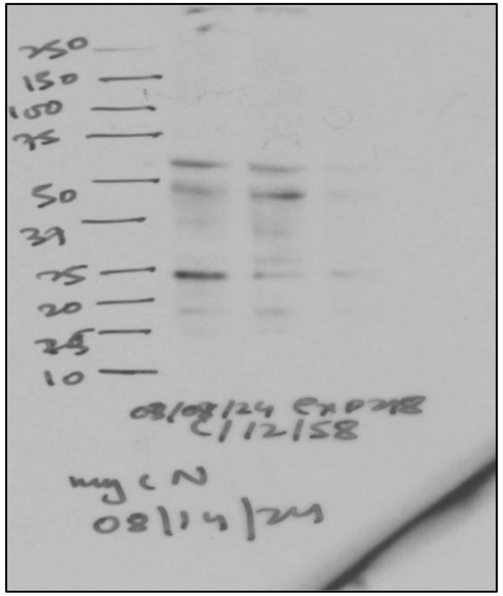

GAPDH

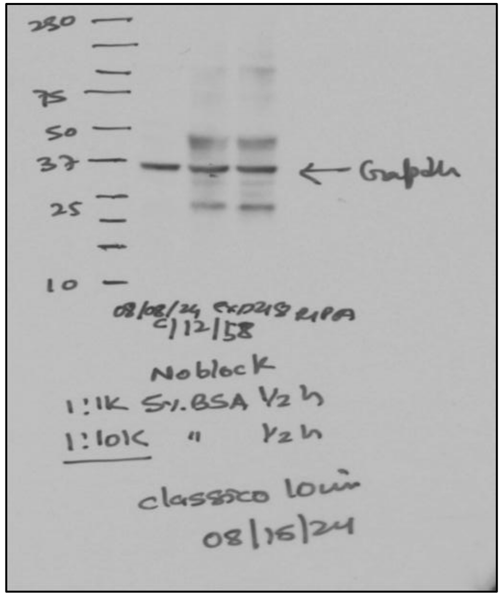

Figure 6d **vehicle/1215/5800**

pBRD4

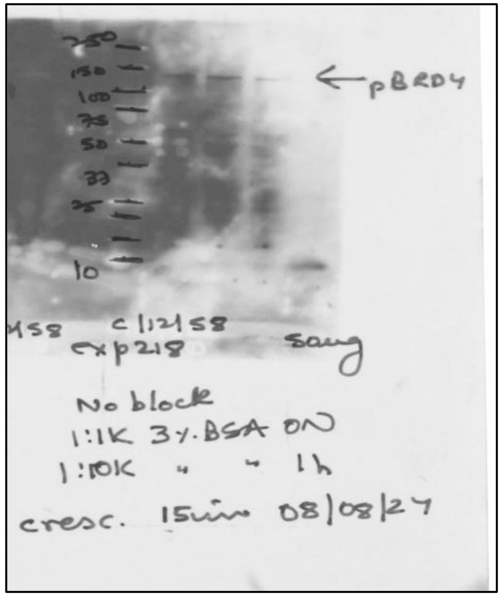

BRD4

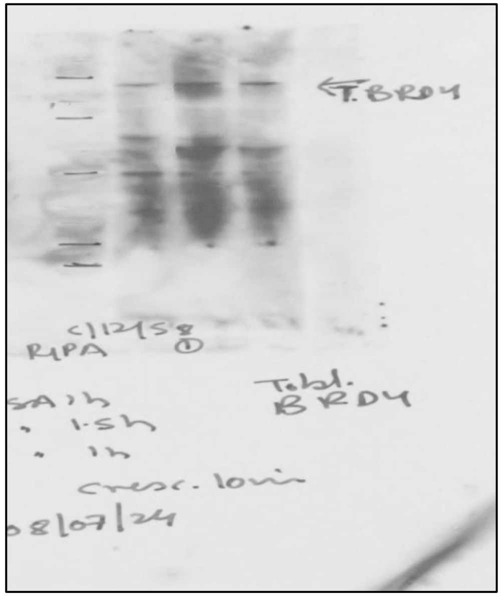

GAPDH

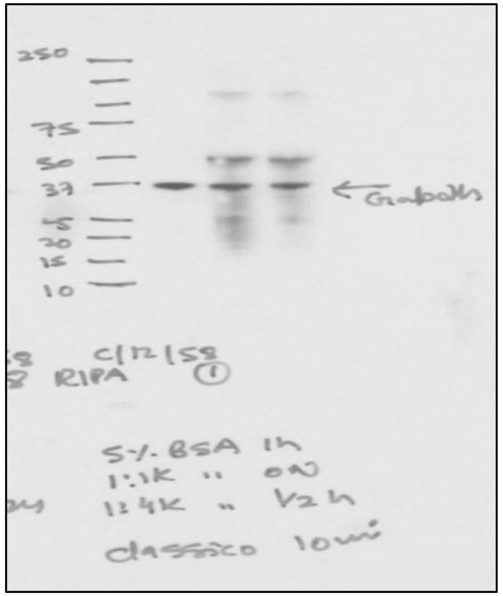

Figure 6d vehicle/1215/5800 SK-N-BE(2)

H3K27ac H3

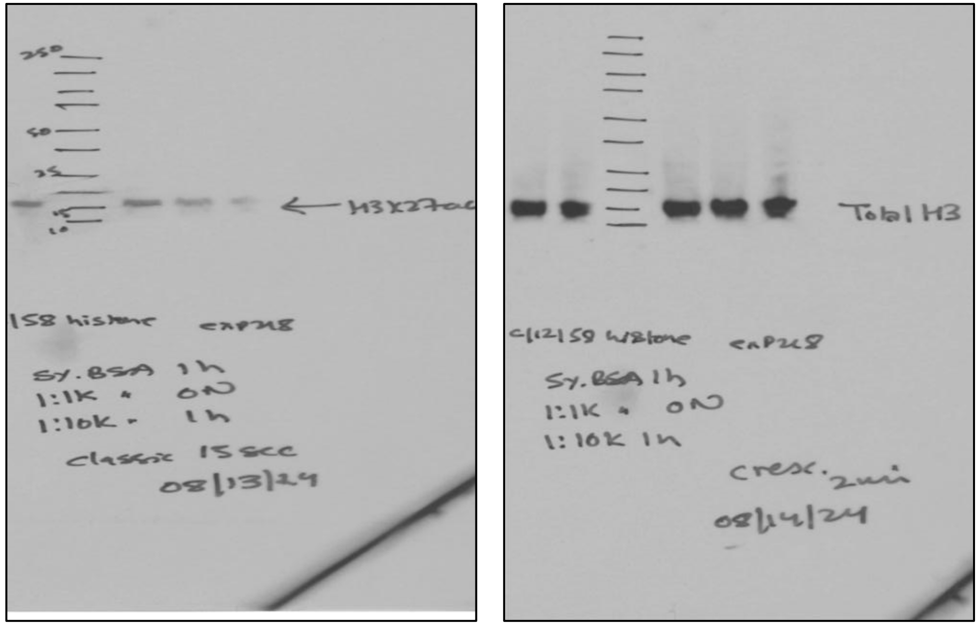

Figure S2a

SK-N-AS SK-N-BE(2) SHEP WAC

MYCN

β-actin

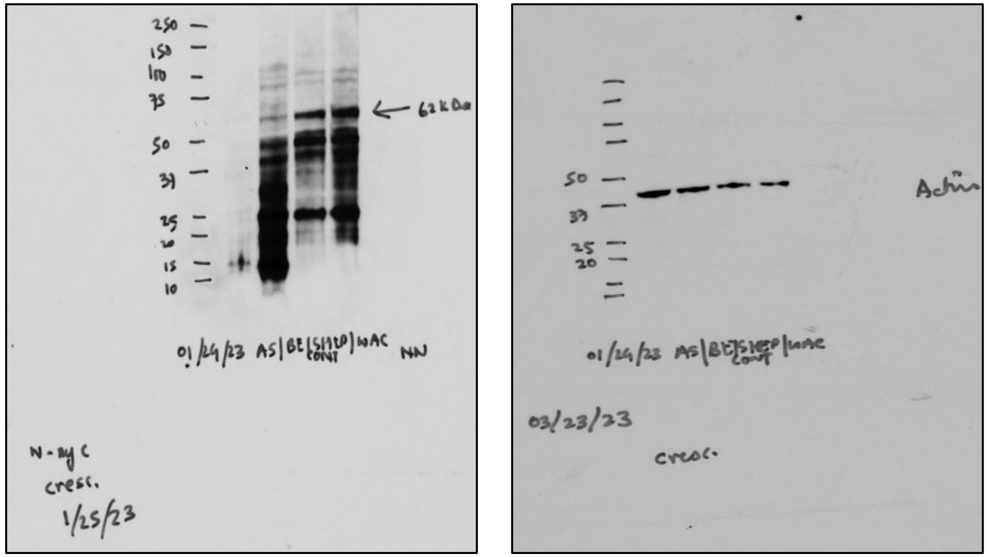

Figure S2b **COA6**

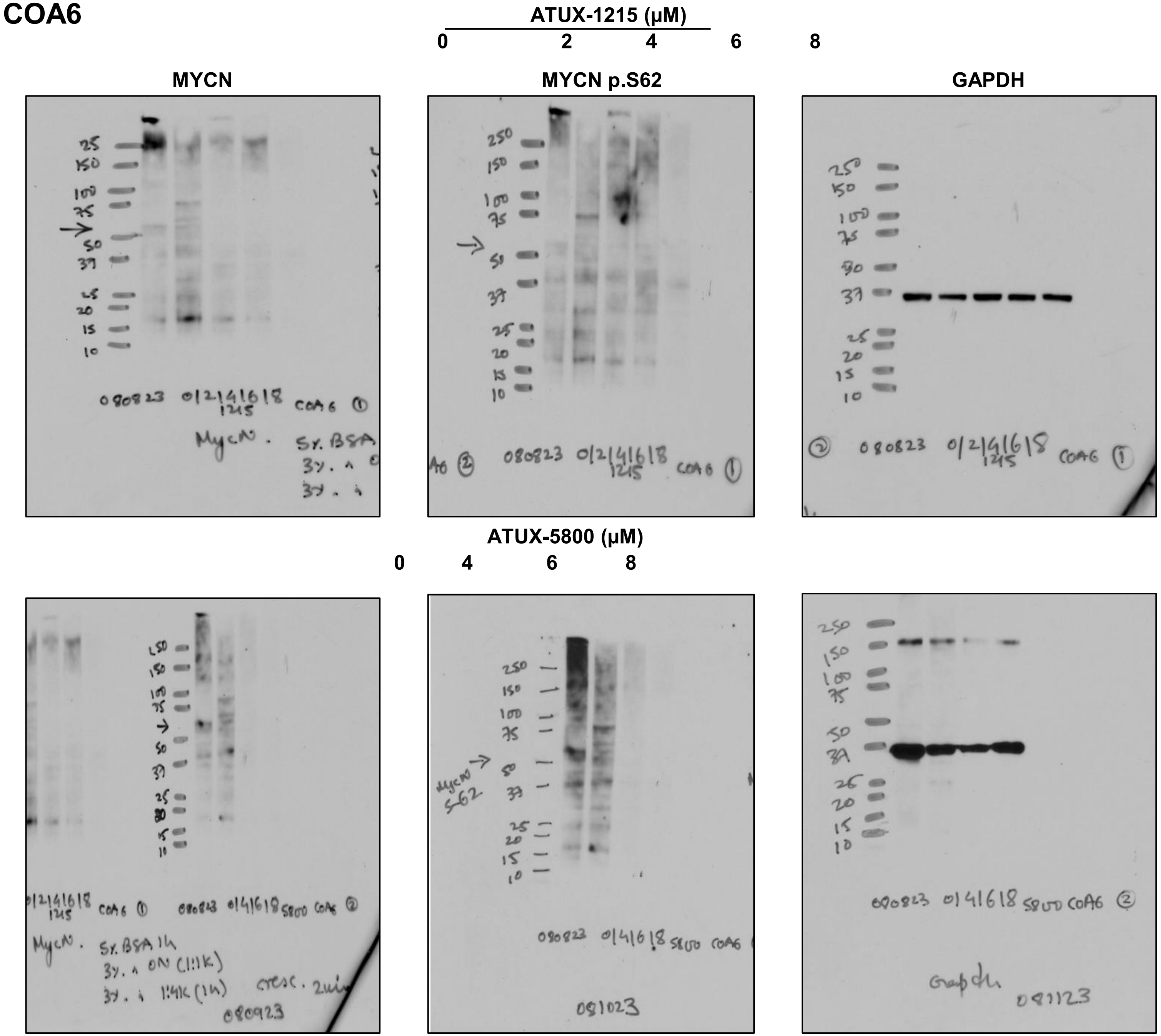

Figure S2c

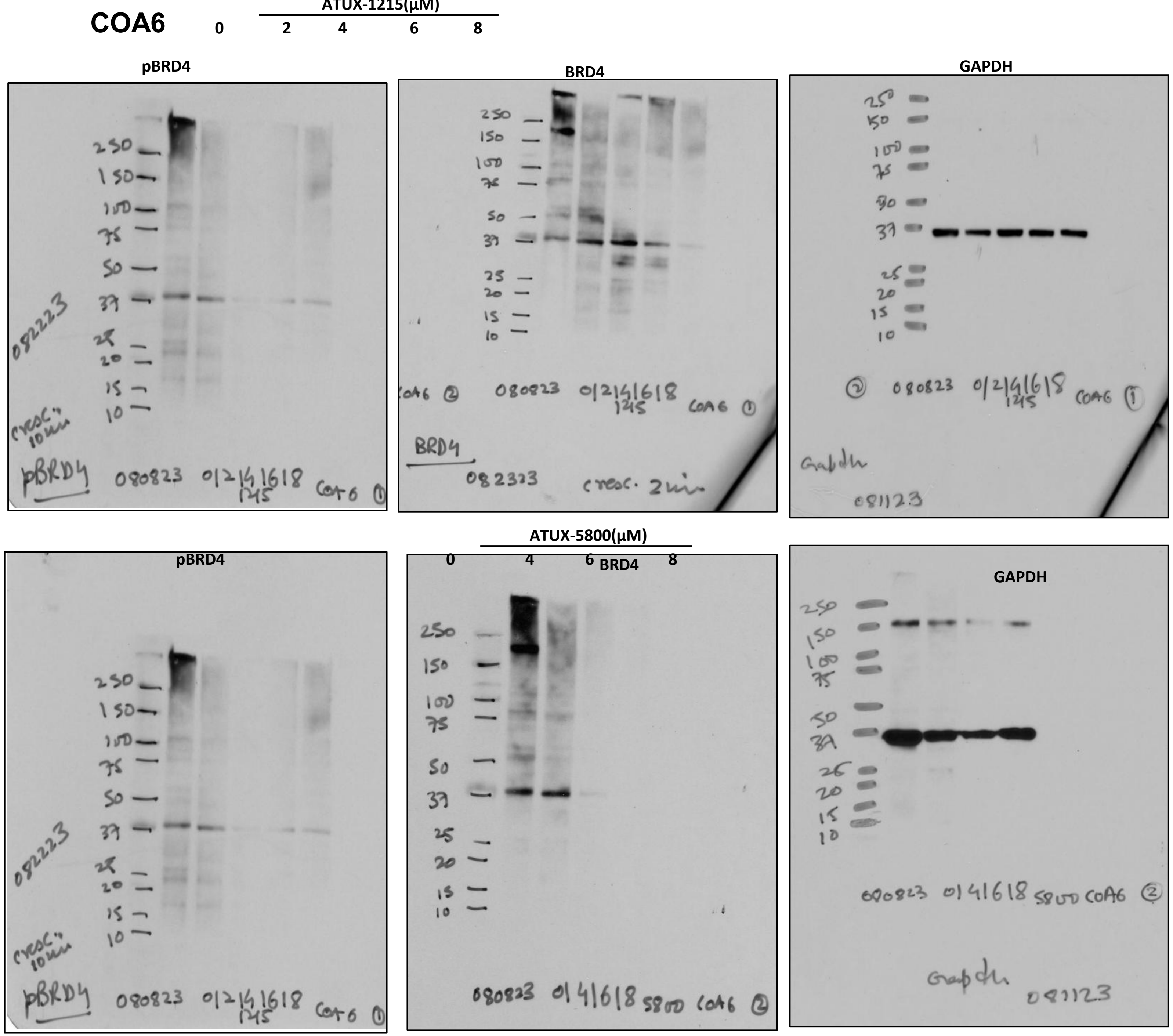

COA6
ATUX-1215(μM)
0
2
4
6
8
pBRD4
BRD4
GAPDH
ATUX-5800(μM)
0
4
6
8
pBRD4
BRD4
GAPDH

Figure S2d

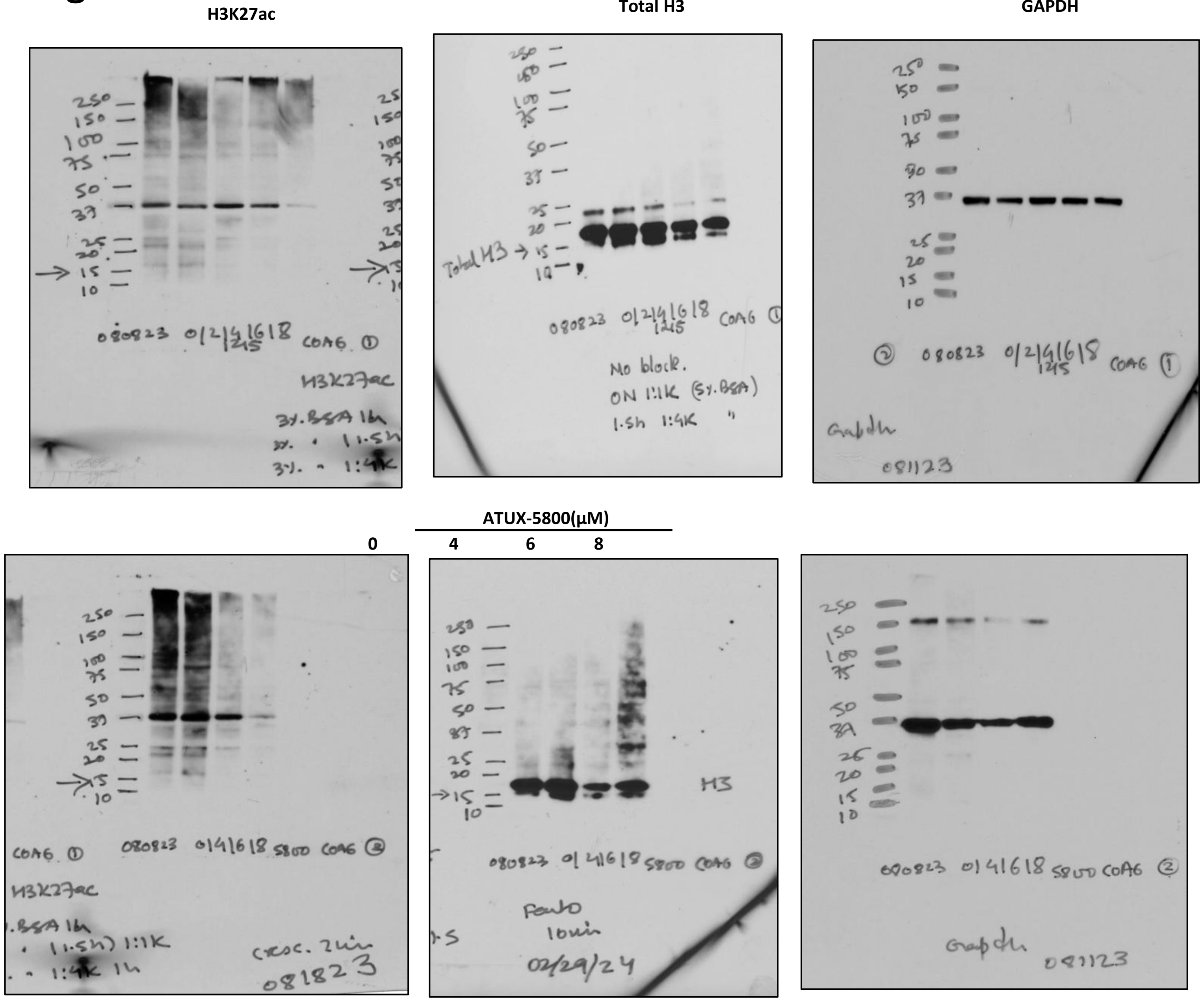
COA6
ATUX-1215(μM)
0
2
4
6
8
H3K27ac
Total H3
GAPDH
ATUX-5800(μM)
0
4
6
8

Figure S2e COA6

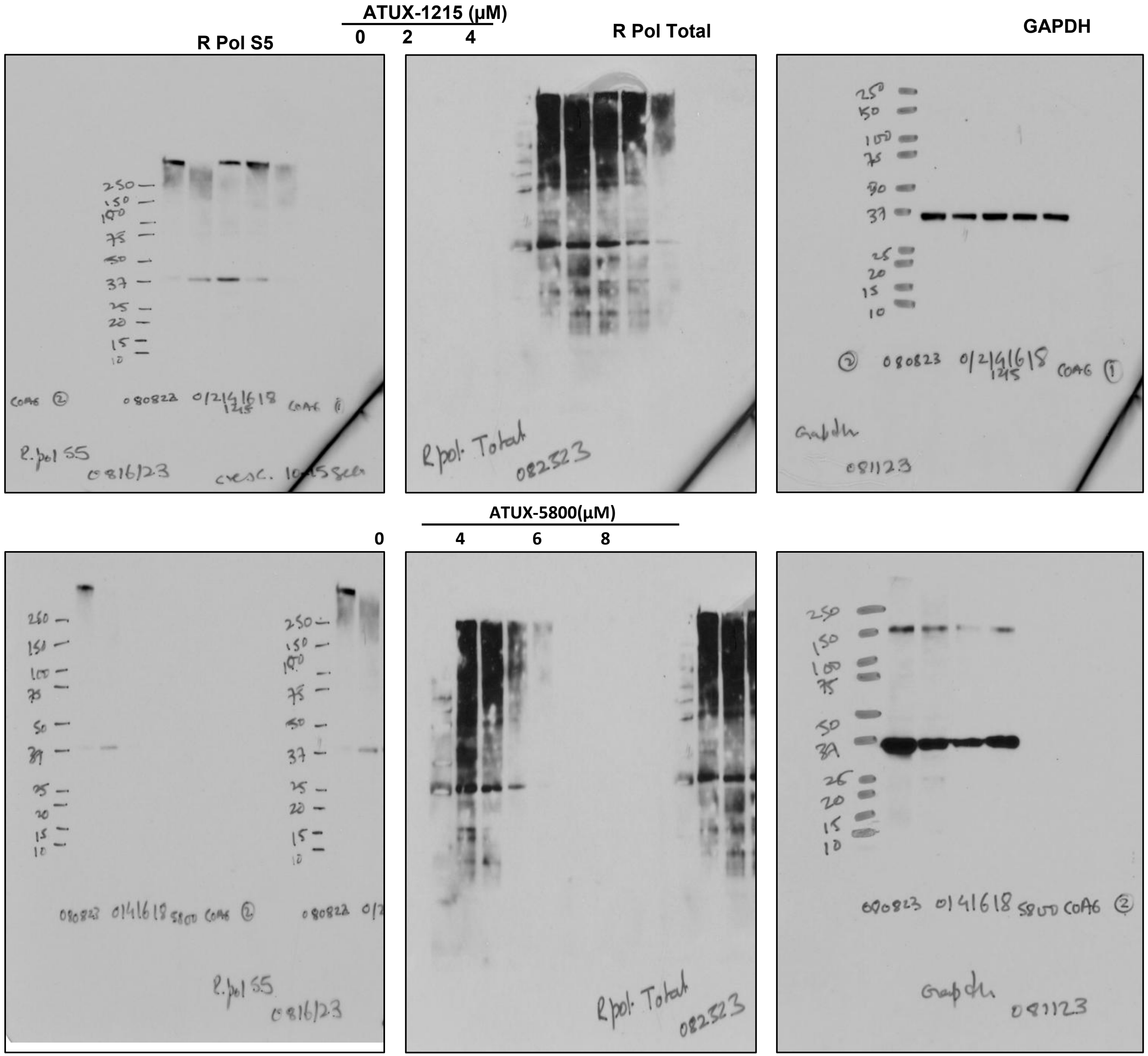

Figure S5a SK-N-AS

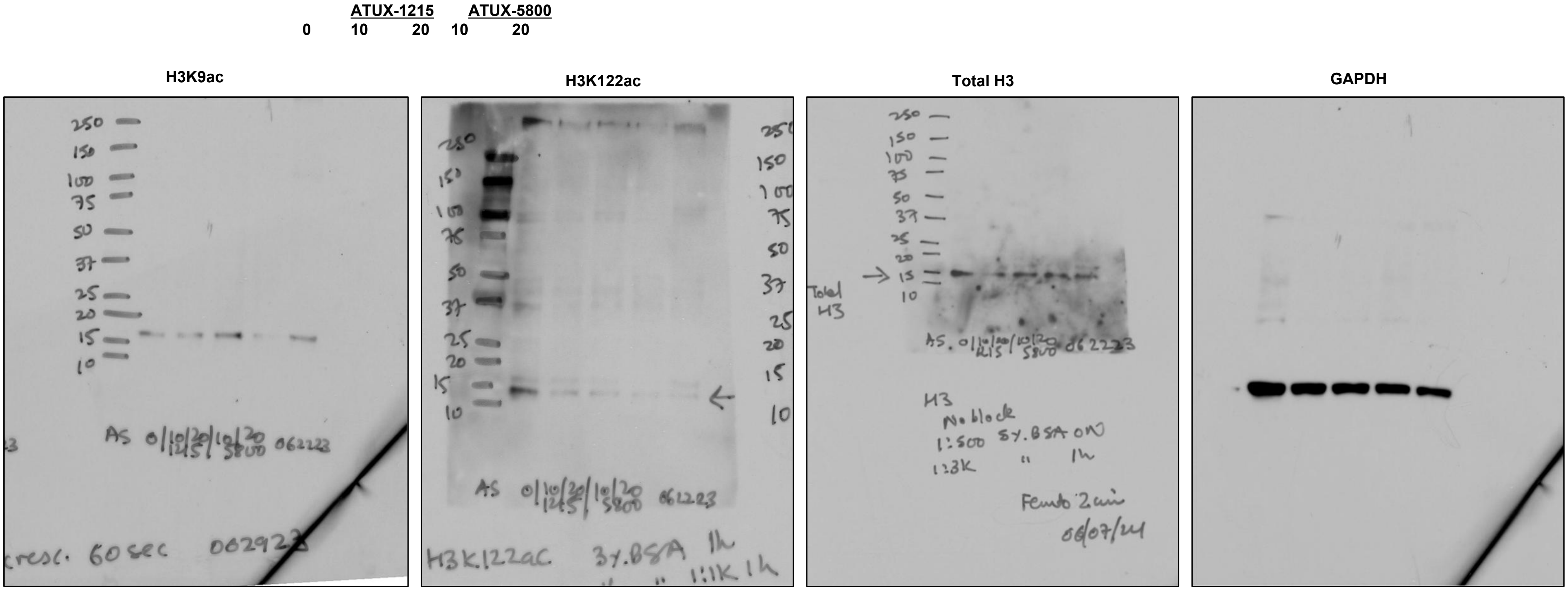

Figure S5b SK-N-BE(2)

	ATUX-1215		ATUX-5800	
0	10	20	10	20

H3K9ac H3K122ac Total H3 GAPDH

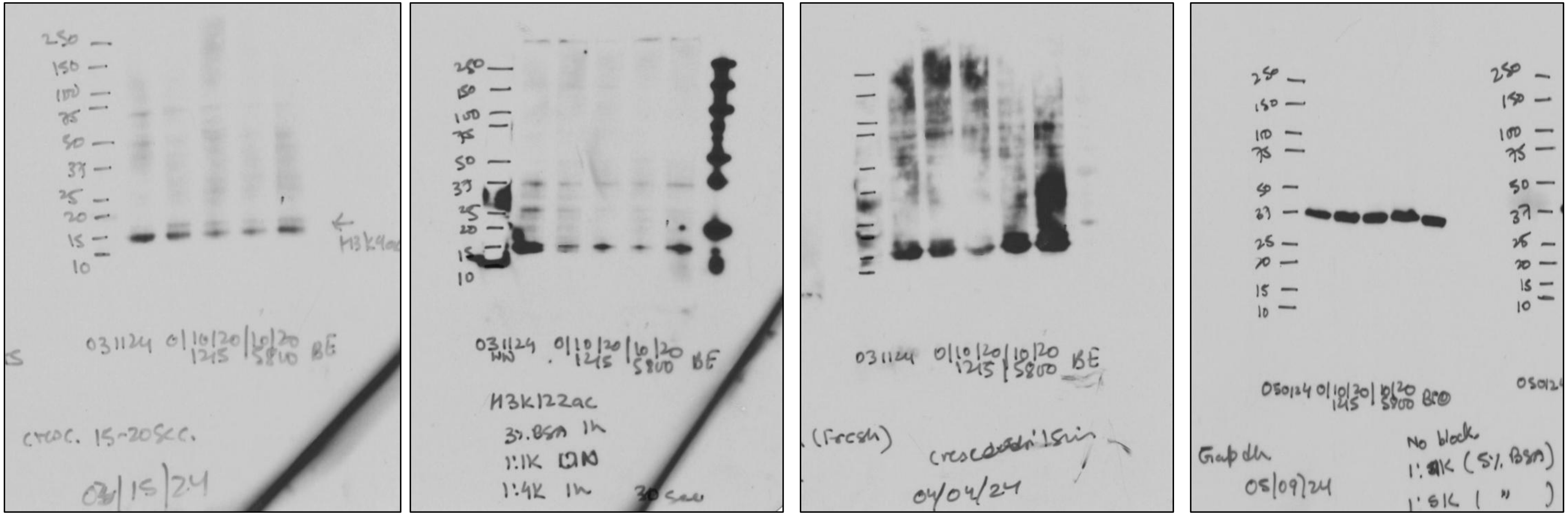

Figure S5c COA6

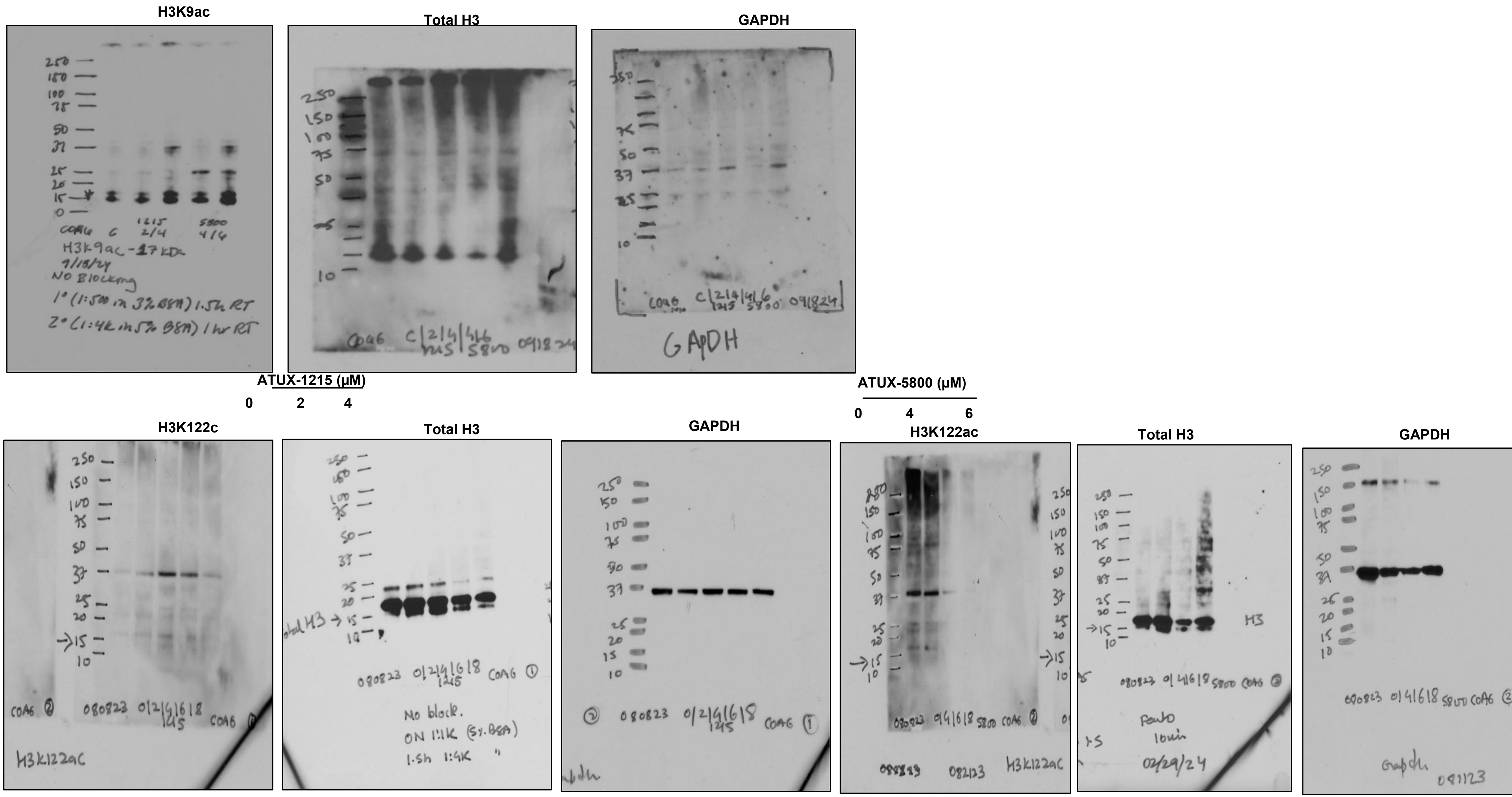

Figure S6a SK-N-AS

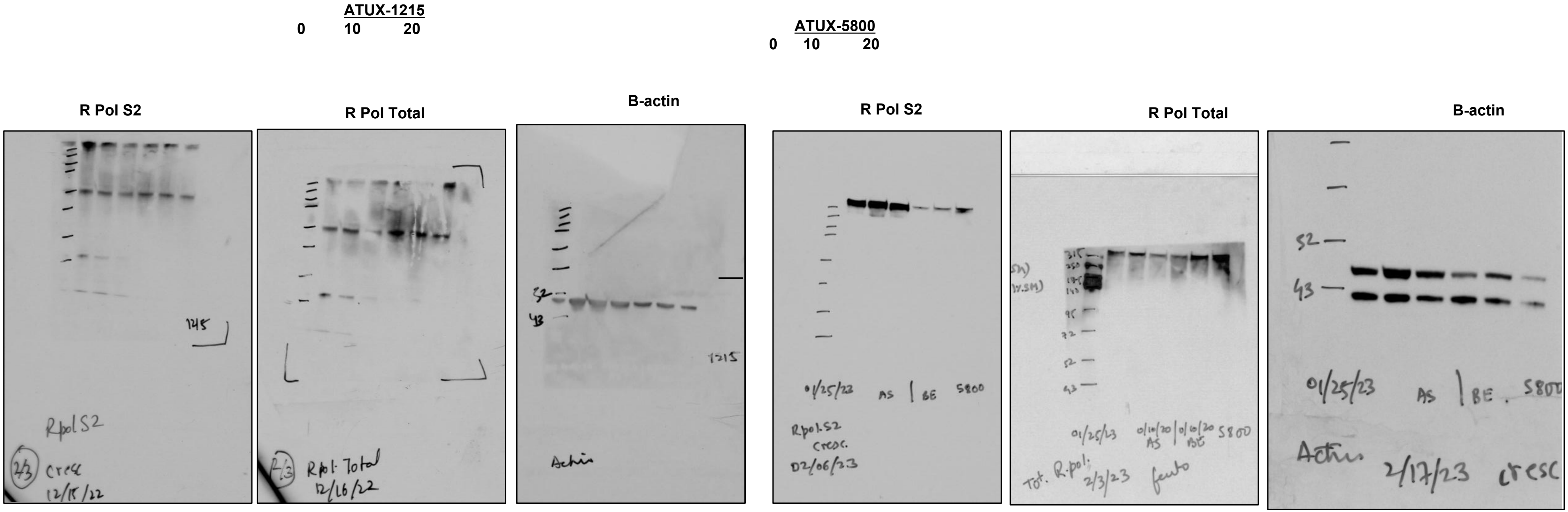

Figure S6b SK-N-BE(2)

ATUX-1215
0 10 20

ATUX-5800
0 10 20

R Pol S2 | R Pol Total | B-actin | R Pol S2 | R Pol Total | B-actin

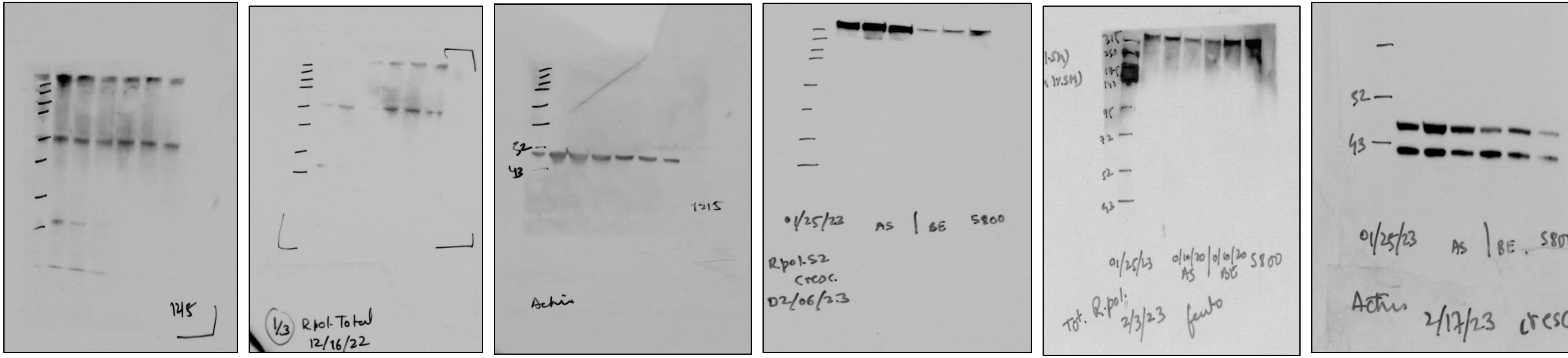

Figure S6c COA6

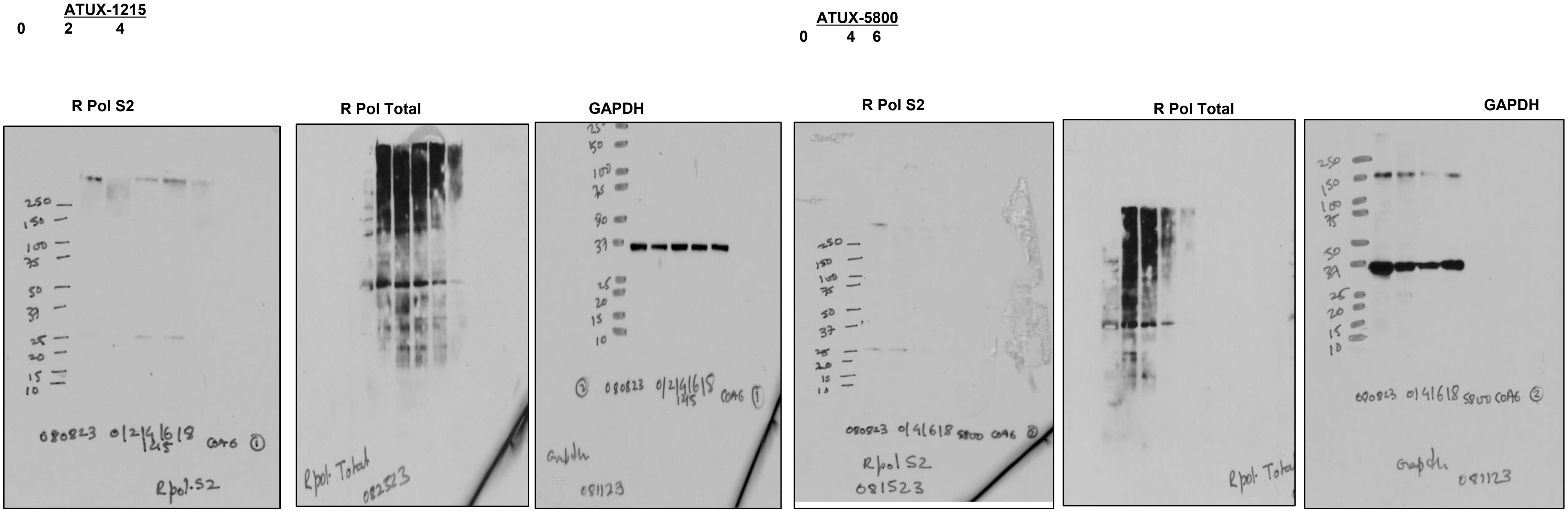

Supplement: Supplementary file 1 — Uncropped blot [file 41419_2025_8253_MOESM1_ESM.pdf]
