# Supplementary material for: PP2A activation targets MYCN in neuroblastoma
Source: Cell Death Dis. 2026 Jan 15;17(1):42. doi: 10.1038/s41419-025-08253-0 (PMC12808165; doi:10.1038/s41419-025-08253-0)
Supplement: Supplementary file 2 — Supplementary figure legends [file 41419_2025_8253_MOESM2_ESM.docx]

**Supplementary Fig. 1 ATUX-1215 and ATUX-5800 activate PP2A and exert cytotoxic effect on NBL cells.**

**a-d,** NBL cell lines and PDX cells were treated with increasing concentrations of ATUX-1215 or ATUX-5800 (SH-EP, WAC(2), 0-15 μM, 24 hr) (COA6, 0-20 μM; COA129, 0-10 μM, 72 hr) and cell viability was measured using alamarBlue assay. Lethal dose 50% (LD50) concentrations were calculated using the dose-response curves (**a-c**). Calculated LD50 data from three independent biologic replicates are presented in tabular form (**d**). The data are presented as mean ± standard error of mean (SEM). Statistical comparison was completed with two-tailed Student’s *t*-test. ∗*p* ≤ 0.05, ∗∗*p* ≤ 0.01, ∗∗∗*p* ≤ 0.001, ∗∗∗∗*p* ≤ 0.0001, *ǂp* ≤ 0.00001. NBL, neuroblastoma; PDX, patient-derived xenograft;1215, ATUX-1215; 5800, ATUX-5800.

### Supplementary Fig. 2 Immunoblotting additional cell lines and antibodies.

**a** Baseline expression of MYCN protein was detected on whole cell lysates of SK-N-AS (*MYCN* non-amplified), SK-N-BE(2) (*MYCN* amplified), SH-EP (*MYCN* non-amplified), and WAC(2) (*MYCN* overexpressed) whole cell lysates. In SK-N-AS cells, the basal level of MYCN protein was below detection on immunoblot (whole cell lysate) compared to the other three cell lines. **b-e** COA6 (*MYCN* amplified PDX, 1 × 10^6^) cells were treated with ATUX-1215 or ATUX-5800 (0-6 μM for 24 hours) and immunoblotting of whole cell lysates completed. ATUX-1215 or ATUX-5800 treatment decreased MYCN total protein expression at higher concentrations and decreased phosphorylation of S62 in PDX COA6 (**b**). Treatment with either of the PP2A activators led to decreased phosphorylation of BRD4 (**c**), reduction in H3K27ac (**d**) and R Pol S5 phosphorylation (**e**) in COA6 PDX cells. β-actin, vinculin, GAPDH, or total H3 were assayed as the loading control.

**Supplementary Fig. 3 BRD4 gene expression negatively correlates with event free and overall survival in *MYCN* amplified and non-amplified NBL patient samples.**

- 1. R2 Genomics Analysis and Visualization Platform was used to evaluate gene expression data from primary human NBL samples (Neuroblastoma-Kocak dataset (GSE45547, n=649). Kaplan-Meier curves were generated based on *BRD4* expression and event-free or overall survival probability in *MYCN* non-amplified and amplified patient groups. There is a negative correlation between *BRD4* expression and event-free (*left panels*) or overall (*right panels*) survival in *MYCN* non-amplified (**a**) and amplified patient samples (**b**).

### Supplementary Fig. 4 Treatment with ATUX-1215 or ATUX-5800 at lower concentration (10 µM) leads to reduced H3K27 acetylation at *MYCN* promoter.

SK-N-BE(2) cells were treated with ATUX-1215 or ATUX-5800 (10 μM) for 24 hours and processed for ChIP-qPCR. Control SK-N-BE(2) cells show enhanced H3K27ac enrichment at the *MYCN* promoter (Primer 1 and 2) while significant decrease of H3K27ac was observed with ATUX-1215 or ATUX-5800 treatment. Data are normalized to that of IgG control (1.0) and expressed as mean fold change ± SEM. Data are compared with Student’s *t*-test. ∗*p* ≤ 0.05, ∗∗*p* ≤ 0.01.

### Supplementary Fig. 5 PP2A activation affects H3K9 and H3K122 acetylation in NBL.

SK-N-AS, SK-N-BE(2), and COA6 PDX (1 × 10^6^ cells) were treated with ATUX-1215 or ATUX-5800 (SK-N-AS, SK-N-BE(2), 0-20 μM; COA6, 0-6 μM) for 24 hours and immunoblotting was used to detect protein expression (histone acetylation at lysine 9, H3K9ac, or 122, H3K122ac, respectively). In SK-N-AS, H3K9ac was decreased following treatment with ATUX-5800 (10 μM), while H3K122ac was decreased by both compounds (**a**). In SK-N-BE(2), decreased H3K9ac was noted following ATUX-5800 treatment (10 and 20 μM) while H3K122ac had inhibited expression at lower ATUX-1215 concentration (10 μM) and for both concentrations of ATUX-5800 (10 and 20 μM) (**b**). Decreased expression of H3K9ac and H3K122ac was seen in COA6 PDX cells treated with ATUX-1215 or ATUX-5800 (**c**). For immunoblotting, GAPDH or total histone 3 (H3) was assayed as the loading control **(a-c)**.

### Supplementary Fig. 6 ATUX-1215 or ATUX-5800 treatment alters phosphorylation state of RNA Polymerase II C terminal domain (R Pol II CTD).

**a**-**c** SK-N-AS, SK-N-BE(2) and COA6 (1 × 10^6^ cells) were treated with increasing concentrations of ATUX-1215 or ATUX-5800 (SK-N-AS, SK-N-BE(2), 0-20 μM; COA6,0-6 μM) for 24 hours and expression of total R Pol and phosphorylation at S2 was detected using immunoblotting. In SK-N-AS cells, decreased phosphorylation of R Pol S2 was noted at the 20 μM concentration of ATUX-1215 (**a**, *left panel*). In SK-N-BE(2) cells, R Pol phosphorylation at S2 was decreased following 20 μM ATUX-1215 treatment (**b**, *left panels*), while ATUX-5800 reduced phosphorylation of R Pol at S2 at 10 μM (**b**, *right panels*). Phosphorylation of R Pol S2 was decreased by both compounds in the COA6 cells (**c**). β-actin or GAPDH served as loading controls.

### Supplementary Fig. 7 ATUX-1215 treatment delays tumor growth for animals bearing SK-N-BE(2) tumors.

**a, b** SK-N-BE(2) cells (1.5 × 10^6^) were injected into the right flank of female athymic nude mice. Once tumors reached 100 mm^3^, animals were randomized into three groups: Vehicle (n = 7), ATUX-1215 (n = 8, 75 mg/d), and ATUX-5800 (n = 9, 75 mg/d), and treated via oral gavage bid for 21 days. Animals were followed for tumor growth after treatment was completed. Kaplan-Meier survival curves were evaluated with log-rank statistics. Tumor bearing mice treated with ATUX-1215 showed greater median time to reach 2500 mm^3^ (median time = 20 days) compared to vehicle treated (median time=14 days) (**a**). Tumors were evaluated with IHC. Staining for Ki67, a marker of cell proliferation, was decreased in tumors from animals treated with ATUX-1215 or ATUX-5800 compared to vehicle treated controls (**b**). Negative control rabbit IgG staining was included (*inset, lower left, first panel*). Magnification for photomicrographs is 40×.
