# Supplementary figures and images for: PP2A activation targets MYCN in neuroblastoma
Source: Cell Death Dis. 2026 Jan 15;17(1):42. doi: 10.1038/s41419-025-08253-0 (PMC12808165; doi:10.1038/s41419-025-08253-0)

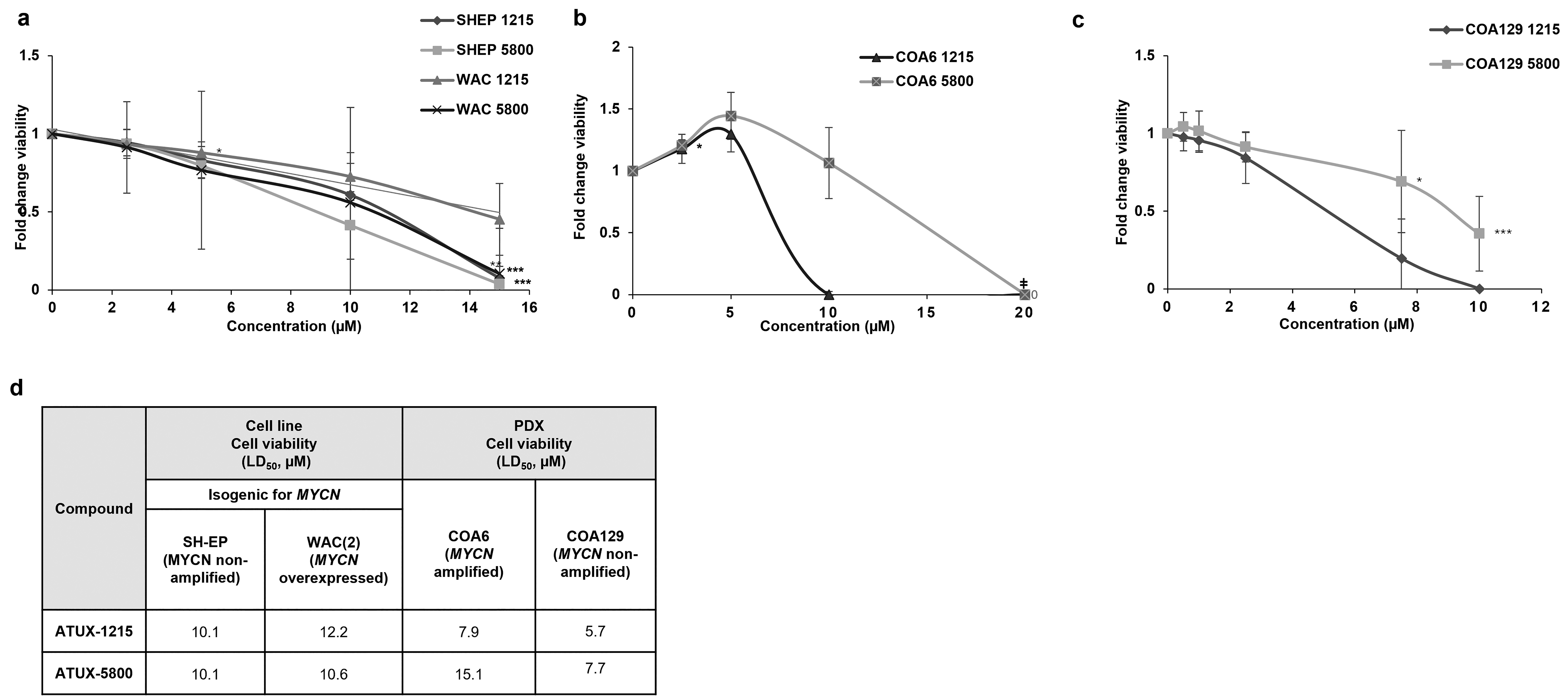

Supplement: Supplementary file 3 — Figure S1 [file 41419_2025_8253_MOESM3_ESM.tif]

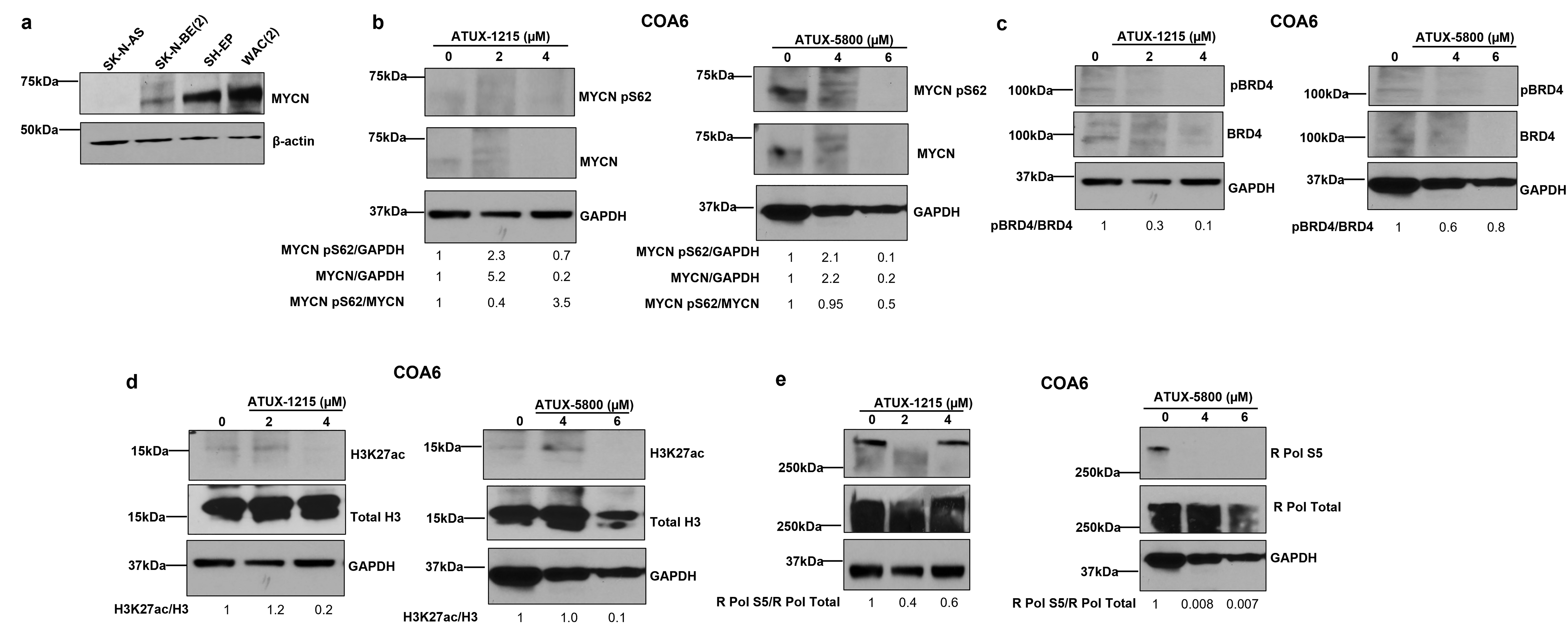

Supplement: Supplementary file 4 — Figure S2 [file 41419_2025_8253_MOESM4_ESM.tif]

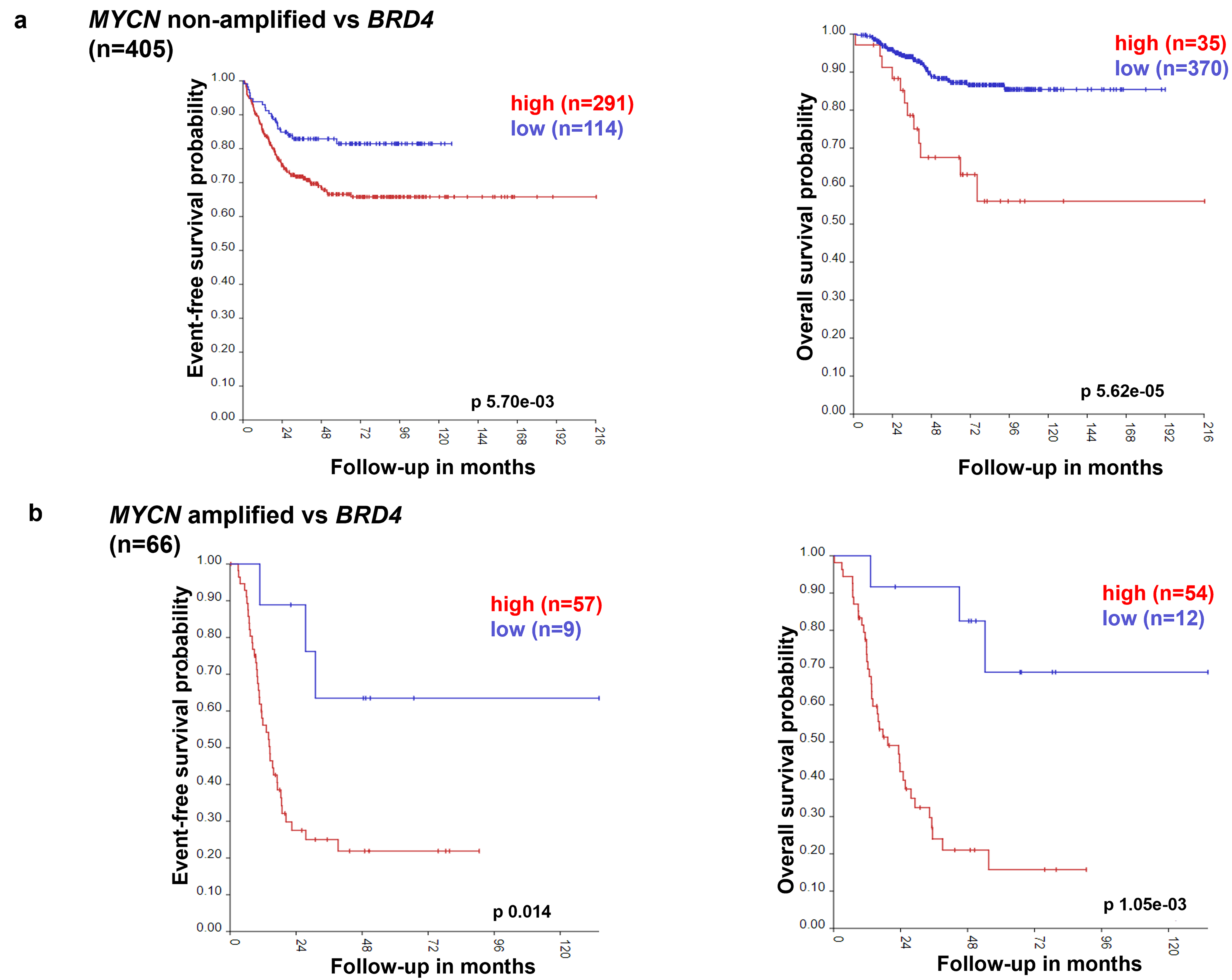

Supplement: Supplementary file 5 — Figure S3 [file 41419_2025_8253_MOESM5_ESM.tif]

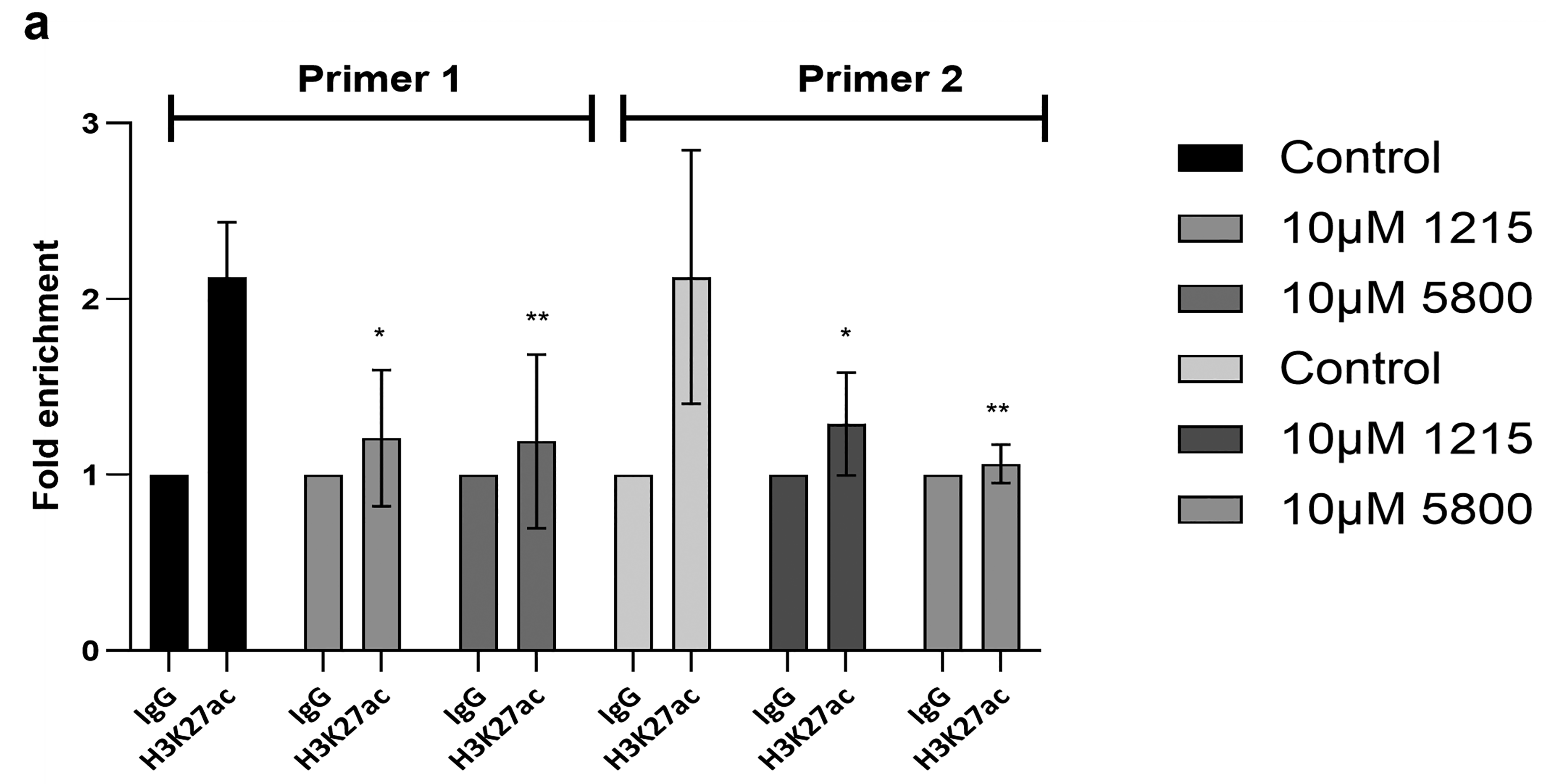

Supplement: Supplementary file 6 — Figure S4 [file 41419_2025_8253_MOESM6_ESM.tif]

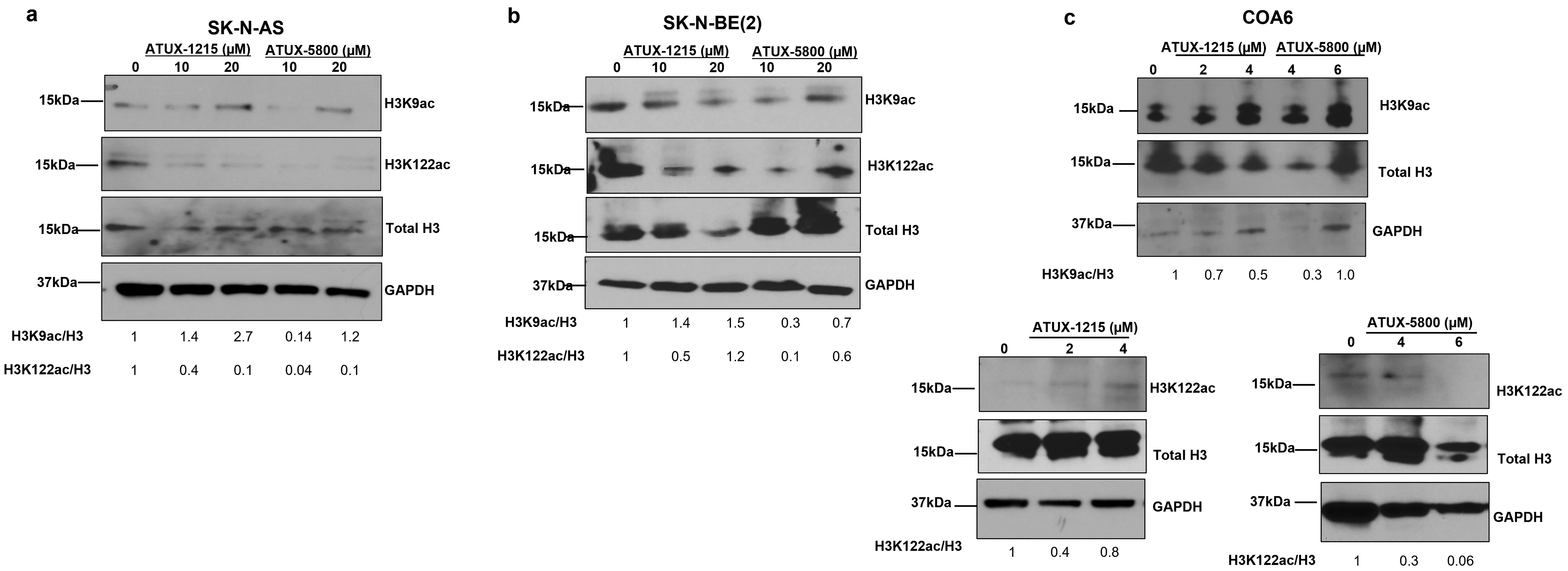

Supplement: Supplementary file 7 — Figure S5 [file 41419_2025_8253_MOESM7_ESM.tif]

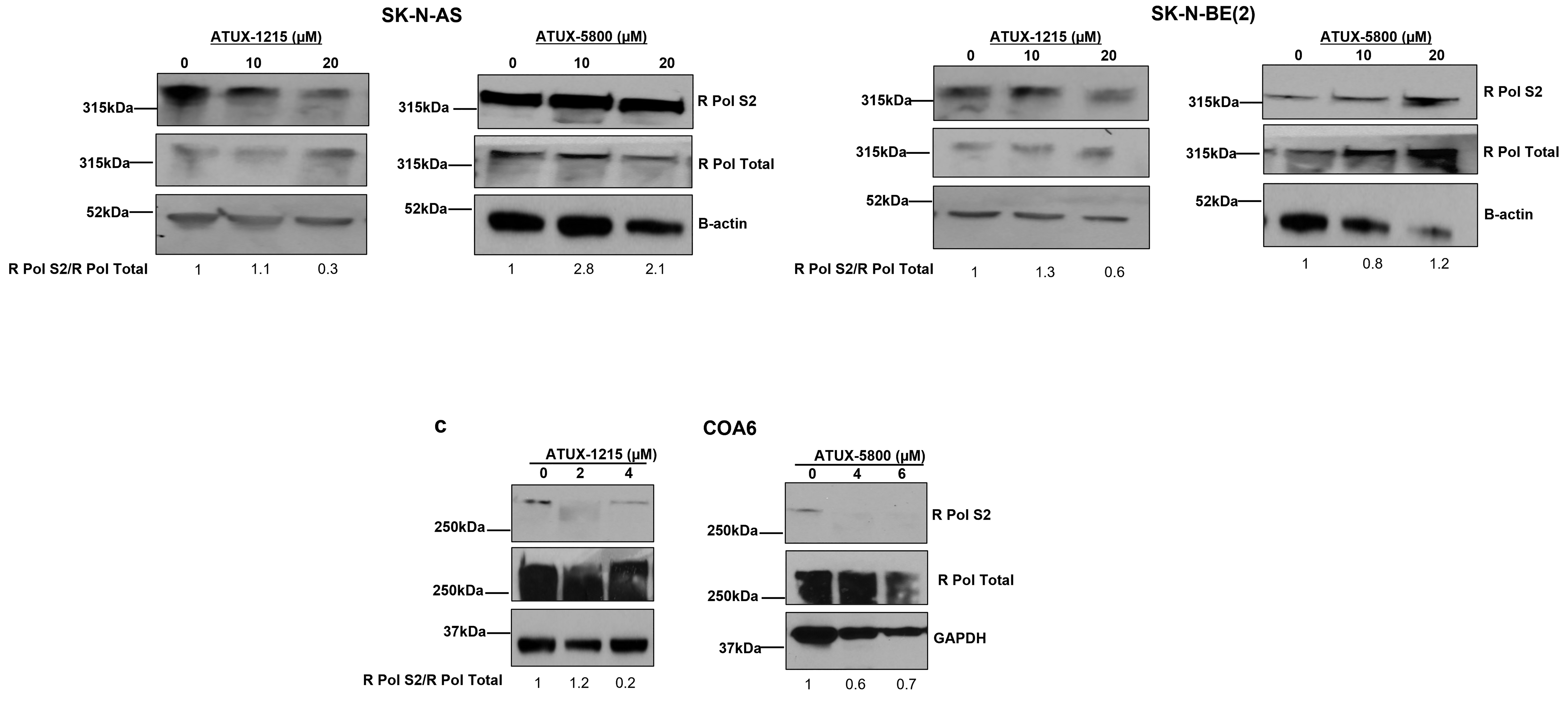

Supplement: Supplementary file 8 — Figure S6 [file 41419_2025_8253_MOESM8_ESM.tif]

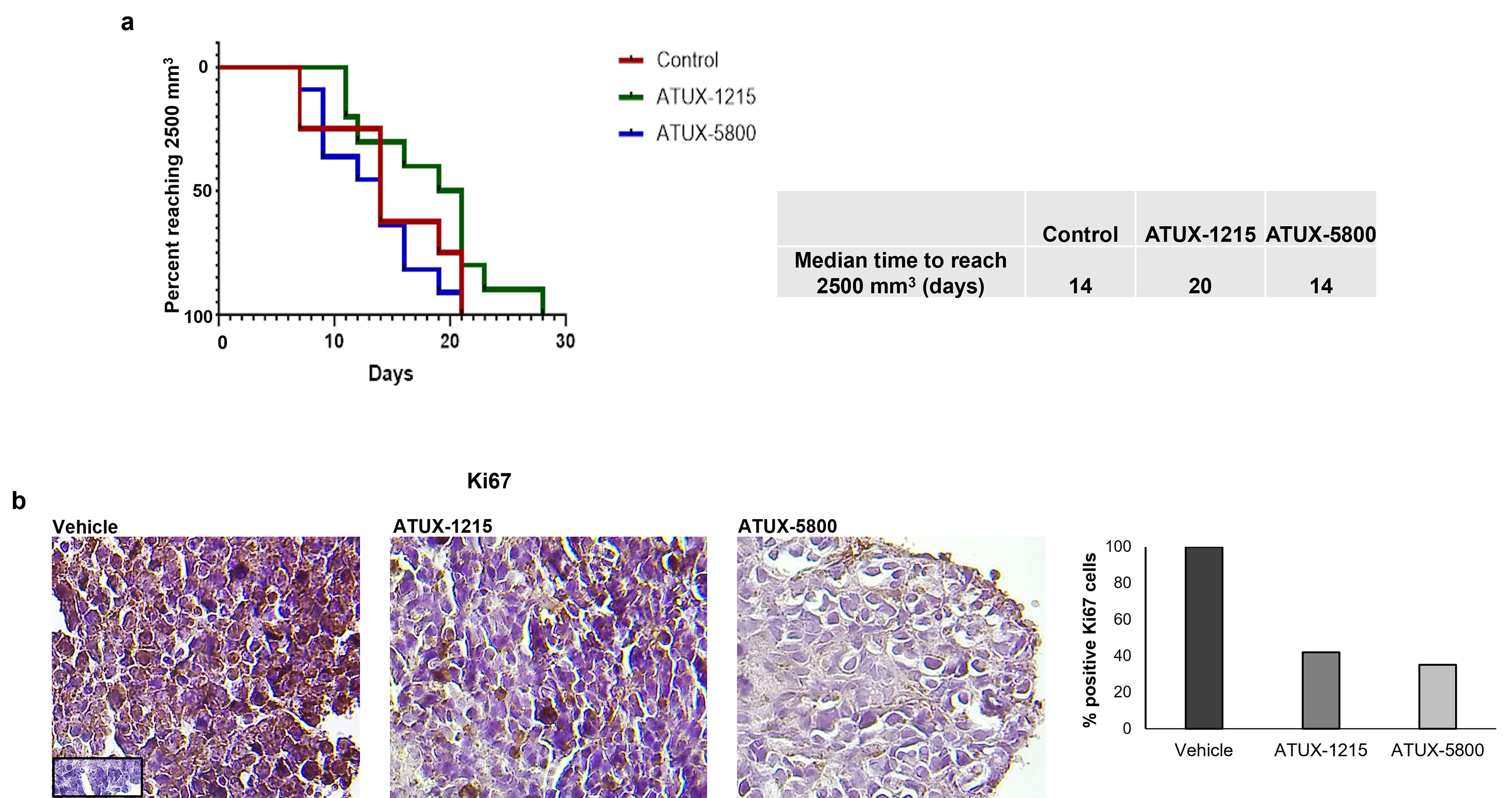

Supplement: Supplementary file 9 — Figure S7 [file 41419_2025_8253_MOESM9_ESM.tif]
